# A 10× continuously zoomable metalens system with super-wide field of view and near-diffraction–limited resolution

**DOI:** 10.1515/nanoph-2025-0399

**Published:** 2025-11-24

**Authors:** Wangzhe Zhou, Shaoqi Li, Yiyi Li, Zongyuan Chen, Man Yuan, Fen Zhao, Yutai Chen, Huan Chen, Zhaojian Zhang, Jiagui Wu, Junbo Yang

**Affiliations:** College of Science, 58294 National University of Defense Technology, Changsha 410073, China; School of Artificial Intelligence, Chongqing University of Technology, Chongqing 401135, China; School of Physical Science and Technology, Southwest University, Chongqing 400715, China

**Keywords:** Moiré metalens, continuous zoom imaging, wide field-of-view, near-diffraction–limited imaging

## Abstract

Moiré metalens is attractive for imaging applications due to their compact form factor and high zoom ratio. Here, we propose a novel Moiré zoom metalens system that achieves a continuous 10× zoom over a focal length range of 2.2–22 mm at 1,064 nm, while extending the full field of view up to 93°. A variable aperture, capable of axial translation, is introduced to jointly suppress aberrations and maintain a large aperture size with f-numbers ranging from 2.5 to 7.5. The system delivers near-diffraction–limited imaging resolution across the entire zoom and field-of-view range, with Strehl ratios exceeding 0.9. This level of performance is comparable to commercial optics and is rarely reported in metalens-based zoom systems. Remarkably, the total optical volume is only ∼4.2 × 32 mm, underscoring its potential for miniaturized imaging. Furthermore, we establish an integrated design and validation pipeline that strategically combines geometric optics, scalar diffraction, and vectorial electromagnetic theory. This multi-theory approach provides an efficient and generalizable pathway for the development of high-performance metalens systems.

## 1 Introduction

Metalenses based on the generalized Snell’s law have emerged as promising candidates to transcend the limitations of conventional optical components, owing to their compact form factors and versatile phase manipulation capabilities [1], [2]. By tailoring the design and spatial arrangement of subwavelength-scale elements (meta-atoms), diverse functional metalens have been developed and demonstrated across a broad range of applications, such as wavefront sensing [3], [4], vortex beam generation [5], [6], binocular vision [7], [8], beam steering [9], [10], optical tweezers [11], [12], holographic displays [13], [14], and optical analog computing [15], [16]. In the field of imaging, metalens have likewise shown immense potential, enabling advances in polarization imaging [17], achromatic focusing [18], [19], and wide-field imaging [20], [21]. However, a major limitation of most current metalens is their fixed focal length, which poses challenges for compact zoom optics demanded in platforms such as smartphones, UAVs, and portable medical devices. While several metalens-based zooming strategies have been proposed, each suffers from specific trade-offs. A straightforward approach, akin to conventional optics, involves varying the axial distance between two metalenses to alter the effective focal length [22], [23]. Alternatively, Alvarez metalens achieve zooming by introducing lateral displacement between two overlapping phase profiles [24], [25]. Nevertheless, both approaches require additional space for mechanical movement, which undermines the intrinsic compactness of metalens. Other routes such as mechanical stretching of flexible substrates [26], induced phase transitions in functional materials [27], [28], and polarization multiplexing [29] offer elegant solutions but often involve complex control schemes and limited aberration correction capabilities.

The emergence of Moiré metalens offers a compelling pathway toward compact zoom optics. Originally demonstrated with diffractive elements, Moiré lenses enable continuous focal length tuning via relative rotational motion between two lenses [30], [31]. Incorporating metalens with subwavelength structures into Moiré system has further pushed the boundaries of zoom performance. To date, Moiré metalens have demonstrated wide zoom ratios across terahertz, visible, and near-infrared bands [32], [33], [34], [35] and have recently been applied to fluorescence microscopy [36] and ultrasound imaging [37]. Nevertheless, the majority of Moiré metalens designs have been limited to on-axis performance. Imaging quality for off-axis points often degrades significantly or requires additional bulk optics for correction [35], [36]. Thus, enabling wide-field zoom imaging with metalens remains a critical open challenge. The Moiré lens inherently supports tunable quadratic phase distribution, which has been shown to facilitate wide field-of-view (FOV) imaging [38], [39]. However, the quadratic phase is a paraxial approximation of a hyperbolic phase, and thus inherently suffers from spherical aberrations that prevent near-diffraction–limited imaging. Some efforts have been made to optimize the phase profile of Moiré metalens for improved off-axis performance, but the resulting focusing fidelity remains suboptimal [40]. An alternative strategy involves introducing fixed apertures and appropriately designed refractive media of appropriate thickness, which have been shown to effectively balance aberrations in ultra-wide-angle, fixed-focus metalens [41], [42], [43], [44], [45], and may offer a promising route for integration into zoomable metalens systems.

In this work, we propose a continuously zoomable metalens system with a super-wide FOV. The design leverages the fundamental principle of Moiré lenses, augmented by a tunable aperture mechanism. At the design wavelength of 1,064 nm, rotating the relative angle between the paired metalenses from 0° to 180° enables continuous focal length tuning from 2.2 mm to 22 mm. At the wide-angle end, the system achieves a maximum full FOV of 93° with an f-number as low as 2.5. At the telephoto end, the adjustable aperture increases the entrance pupil diameter and translates axially to maintain a minimum f-number of 7.5. The system maintains near-diffraction–limited performance across the entire zoom range, with a Strehl ratio exceeding 0.9 throughout. Furthermore, we present a comprehensive design and validation workflow that integrates multiple optical theories for mutual verification. Macroscopic system parameters are optimized using geometric optics, while imaging performance is rigorously evaluated by combining scalar and vector diffraction theories.

## 2 Design and principle

**Figure 1:**
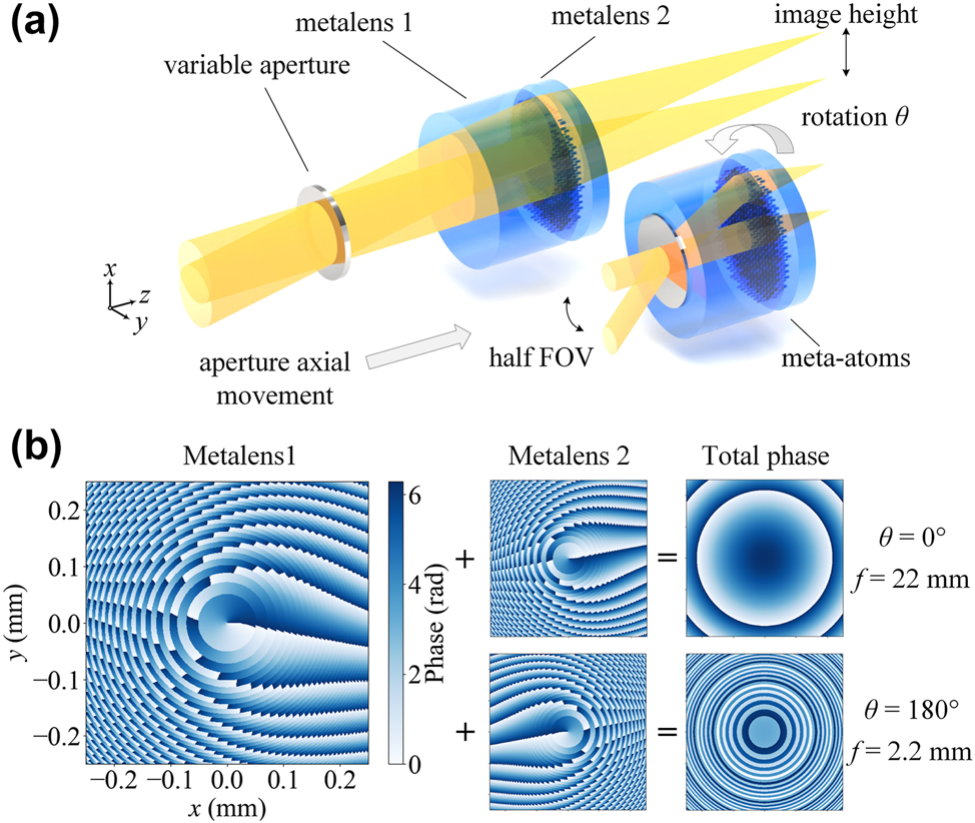
Wide FOV Moiré metalens zoom system and its phase modulation mechanism. (a) Schematic of the wide FOV Moiré metalens zoom system. The key components include a variable aperture, metalens 1 (ML1), and metalens 2 (ML2). Phase-modulating meta-atoms are patterned on the back surface of ML1 and the front surface of ML2. (b) Phase profiles in the central region of ML1’s back surface and ML2’s front surface. By rotating ML2 to different relative angles, the combined phase distribution formed by the two surfaces is modulated accordingly, enabling continuous tuning of the focal length.

Figure 1(a) illustrates the operational principle of the proposed wide field-of-view (FOV) Moiré zoom metalens system, which comprises two metalenses and a variable aperture. The first metalens (ML1) is relatively thick and has a periodic array of meta-atoms on its rear surface. The second metalens (ML2) is thinner, and its front surface structured to interact with ML1, thereby generating the Moiré effect. Transitioning from the telephoto to the wide-angle state involves three coordinated operations: (i) rotating ML2 counterclockwise (from the *y*-axis toward the *x*-axis), (ii) translating the aperture forward along the +*z* direction, and (iii) reducing the aperture diameter. As illustrated in Figure 1(b), in the absence of relative rotation, the complex transmission functions of ML1’s back surface and ML2’s front surface are defined in polar coordinates $\left(\rho ,\varphi \right)$ as:

(1) $$\begin{array}{c} \begin{array}{c} T_{1}=\exp\left[i\text{round}\left(a\rho^{2}\right)\varphi \right]\exp\left(-\frac{i\pi \rho^{2}}{2f_{offset}\lambda }\right),\\ T_{2}=\exp\left[-i\text{round}\left(a\rho^{2}\right)\varphi \right]\exp\left(-\frac{i\pi \rho^{2}}{2f_{offset}\lambda }\right), \end{array} \end{array}$$

where *a* is a design constant, *f*_offset_ is the offset focal length when *θ* = 0, *θ* denotes the in-plane counterclockwise rotation angle of ML2 along the *z*-axis, and *λ* is the design wavelength in vacuum. The $\text{round}\left(\cdot \right)$ function performs upward rounding to ensure that the phase term $\text{round}\left(a\rho^{2}\right)\varphi$ remains 2π-periodic. This avoids the emergence of spurious angular sectors in the Moiré pattern upon rotation [30]. Under the ideal condition where the spacing between the two metalenses is negligible, the combined transmission function *T*_total_ of the metalens pair can be expressed as the product of *T*_1_ and *T*_2_ (x) (the detailed derivation can be found in Section S1 of the Supplementary Material):

(2) $$\begin{array}{c} T_{\text{tolal}}=T_{1}T_{2}=\exp\left[i\text{round}\left(a\rho^{2}\right)\theta \right]\exp\left(\frac{i\pi \rho^{2}}{f_{offset}\lambda }\right). \end{array}$$

This expression describes a typical metalens with a quadratic phase profile. When *θ* = 0, the transmission reduces to a pure quadratic phase term $\exp\left(i\pi \rho^{2}/f_{o\mathrm{ff}set}\lambda \right)$ , which can be tuned by adjusting *f*_offset_ to compensate for the initial phase distribution of the Moiré lens pair. As the rotation angle *θ* increases, the radial phase gradient in *T*_total_ intensifies, resulting in a shorter focal length.

To achieve aberration-free focusing, the output wavefront must form a converging spherical wave. When the incident light is a plane wave propagating along the *z*-axis – corresponding to an object at infinity along the optical axis – the ideal metalens pair should impart a hyperbolic phase profile to the wavefront. Under the paraxial approximation, the hyperbolic phase function can be expanded via a Taylor series into a quadratic form:

(3) $$\begin{array}{c} \Phi_{\text{quad}}\left(\rho \right)=\frac{2\pi }{\lambda }\left[f-f\sqrt{1+{\left(\frac{\rho }{f}\right)}^{2}}\right]\approx \frac{2\pi }{\lambda }\frac{\rho^{2}}{2f}=A\rho^{2}, \end{array}$$

where *A* is the quadratic coefficient. This approximation is valid in the regime where *ρ* ≪ *f*. The benefit of using a quadratic phase is its ability to focus obliquely incident light in a manner similar to that of normally incident light [39], making it advantageous for off-axis performance. However, due to its paraxial nature, a purely quadratic phase inevitably introduces spherical aberrations for large aperture sizes under normal incidence, and coma under oblique incidence. To achieve near-diffraction–limited imaging, we employ an aperture to block peripheral rays and suppress aberrations. However, the aperture size cannot be arbitrarily reduced, as it defines the system’s entrance pupil diameter *D*, which directly determines the f-number (F/#). According to scalar diffraction theory, the full width at half maximum (FWHM) of the resulting Airy disk is given by [46]:

(4) $$\begin{array}{c} FWHM=1.03\lambda \left(\text{F}/\#\right)=1.03\lambda \frac{f}{D}. \end{array}$$

Equation (4) clearly indicates that as the focal length *f* increases, if the pupil diameter *D* remains fixed, the FWHM grows proportionally with the zoom ratio. This leads to a rapid degradation of diffraction-limited resolution at the telephoto end, resulting in a significant loss of high-frequency image information. Therefore, in our design, the aperture diameter is gradually increased with the zoom ratio to maintain high-resolution performance at longer focal lengths. Additionally, we increase the thickness of ML1, which causes obliquely incident rays to refract away from the optical axis inside the substrate. As a result, light rays from different field angles are spatially separated along the radial direction rather than sharing the same region of the metalens. Since the phase gradient of the quadratic phase profile increases linearly with radius, according to the generalized Snell’s law [1], regions closer to the edge possess stronger deflection capabilities. At wide field angles, the metalens must induce larger angular deviations to maintain focus. By jointly optimizing the substrate thickness of ML1 and the axial position of the aperture, we are able to control where rays from various field angles intersect the metalens. This minimizes off-axis aberrations caused by insufficient local focusing power.

**Figure 2:**
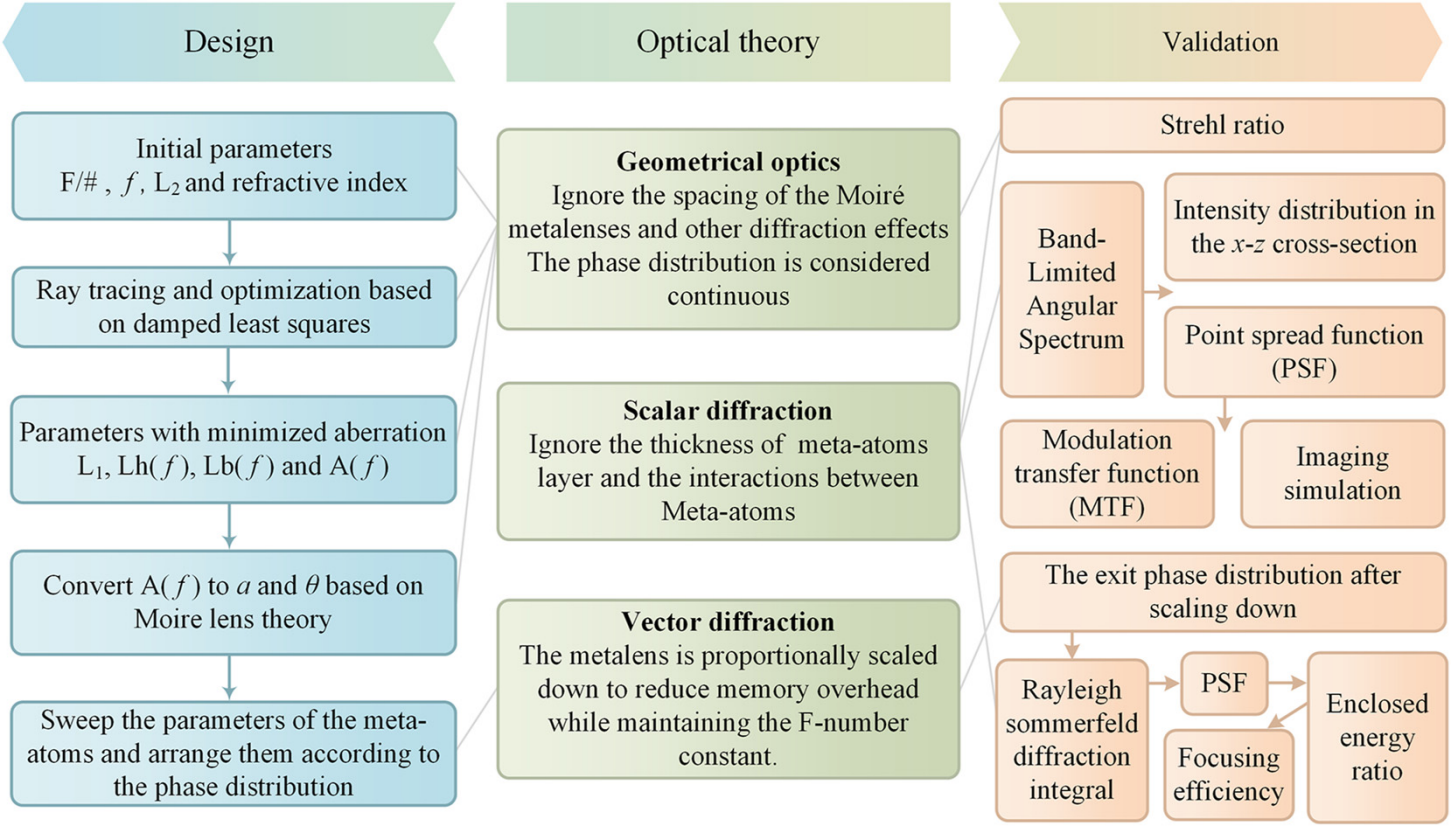
Comprehensive design and validation framework employed in this study. A hybrid strategy integrating geometrical optics, scalar diffraction, and vectorial diffraction is adopted throughout the workflow. Each theory is applied at specific stages according to its strengths, enabling an efficient yet accurate development process for the varifocal metalens system.

Due to the varying degrees of approximation inherent in different optical theories, their computational demands also differ significantly. In this work, we adopt a hybrid design and validation workflow that strategically integrates multiple optical theories, each applied according to its strengths at different stages of the process, as illustrated in Figure 2.

**Figure 3:**
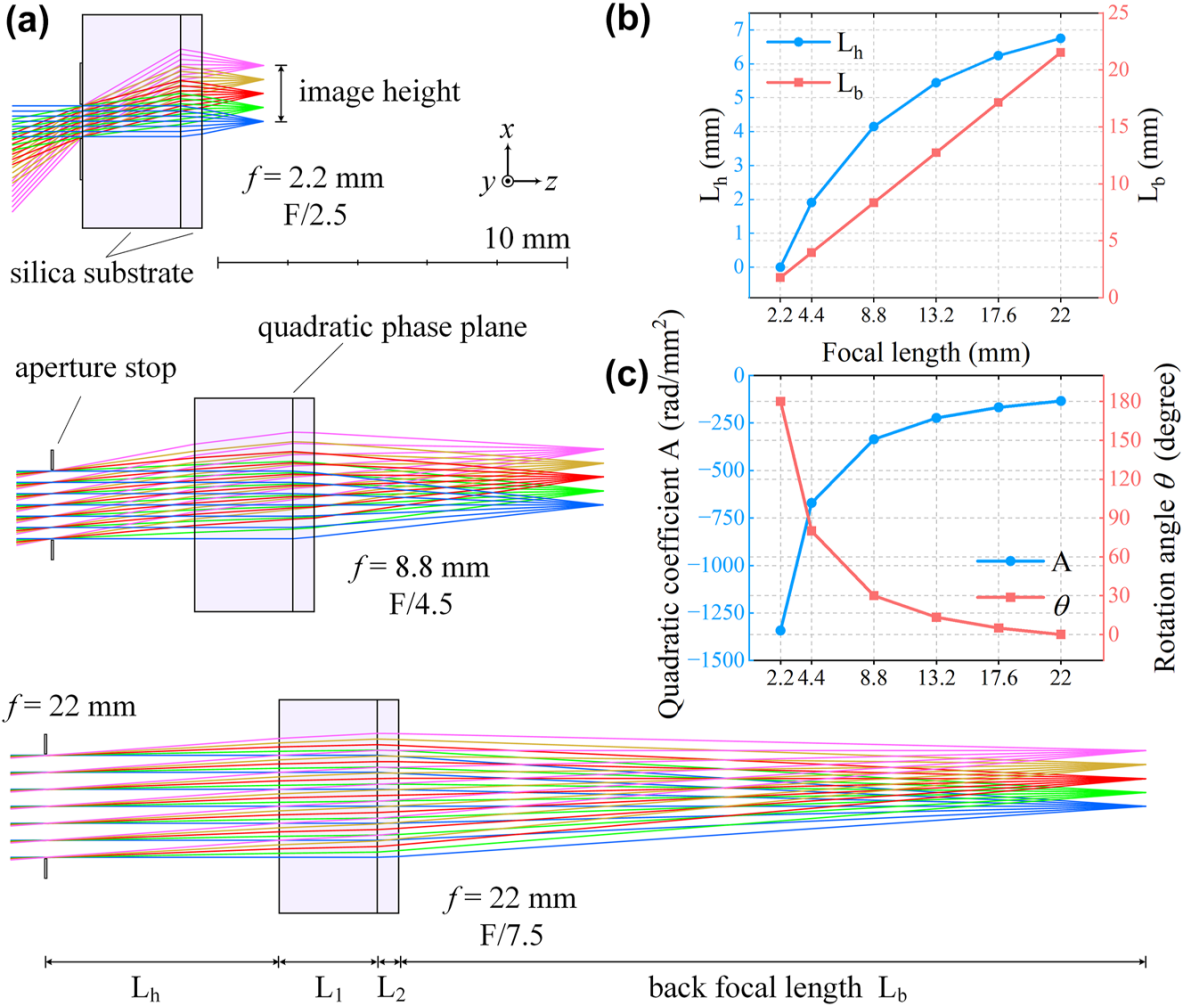
Ray tracing analysis and parametric optimization of the wide FOV Moiré metalens zoom system. (a) Schematic of geometric ray tracing for the wide FOV Moiré zoom system, where the combined effect of ML1’s back surface and ML2’s front surface is approximated by a quadratic phase plane. (b) Optimized relationships between the aperture distance *L*_*h*_, back focal length *L*_*b*_, and system focal length. (c) Optimized quadratic coefficient *A*, and corresponding rotation angle *θ* of ML2 for different focal lengths.

Geometric optics, which models light as rays, offers the fastest computation speed. This efficiency makes it highly suitable for iteratively refining design parameters during early-stage development. To apply this theory to metalens design, two primary assumptions are made. First, the distance between ML1 and ML2 is neglected, and their combined optical effect is treated as a single phase profile described by Equation (3), corresponding to the quadratic phase surface shown in Figure 3(a). Second, diffraction effects are ignored, and only the refractive index of the medium is considered. Light refraction through the quadratic phase is governed by the generalized Snell’s law. Using ray tracing and the damped least-squares algorithm, we optimize the macroscopic parameters of the zoom metalens system. Subsequently, the Strehl ratio is employed as a preliminary metric to evaluate the system performance. It is calculated based on the optimized continuous phase profile, specifically obtained by performing a fast Fourier transform of the optical path differences at the exit pupil.

Given that meta-atoms have subwavelength dimensions, rigorous vectorial diffraction theory is required for accurate modeling. After selecting eight optimized meta-atoms capable of spanning a full 0–2π phase shift, scalar diffraction simulations are conducted using the corresponding discretized phase profile. Scalar diffraction allows simulations at the full physical size of the metalens but neglects meta-atom height and interelement coupling. Scalar diffraction allows visualization of the focused beam distribution in both the *x*–*y* and *x*–*z* planes, with the *x*–*y* cross section at the focal plane representing the system’s point spread function (PSF). This PSF is then used to compute the modulation transfer function (MTF) and to simulate realistic imaging performance.

Vectorial diffraction provides an accurate description of electromagnetic wave behavior but requires substantial computational resources. To maintain the same diffraction-limited resolution as the original full-scale system, the metalens system is uniformly scaled down to the micrometer scale while keeping the f-number constant. Using finite-difference time-domain (FDTD) simulations, the output phase profile is obtained, which is then used in the Rayleigh–Sommerfeld diffraction integral to compute the point spread function (PSF). Analysis of the PSF yields the encircled energy ratio and allows for the evaluation of focusing efficiency.

Together, these three optical theories cross-validate one another, and through multiple performance metrics, demonstrate that our metalens design achieves diffraction-limited resolution across the entire zoom and FOV range.

## 3 Optimization and verification

Following the design principles described above, the first step is to determine three key parameters: the substrate thickness of ML1 $\left(L_{1}\right)$ , the axial distance from the aperture to the front surface of ML1 $\left(L_{h}\right)$ , and the aperture diameter. In recent years, the use of Zemax OpticStudio to assist in metalens system design has been widely reported [43], [45], [46], [47], [48]. Here, we employ Zemax to optimize the parameters of the wide FOV Moiré metalens zoom system. We constrain the focal lengths to *f* = 2.2, 4.4, 8.8, 13.2, 17.6, and 22 mm at a design wavelength of 1,064 nm. Considering the trade-off between aperture size, aberration suppression, and diffraction-limited resolution, the minimum f-numbers for these focal lengths are set to 2.5, 3.5, 4.5, 5.5, 6.5, and 7.5, respectively, in order to achieve optimal imaging performance. The numerical apertures (NA) corresponding to different focal lengths are 0.196, 0.141, 0.110, 0.091, 0.077, and 0.067, where NA is defined as $\text{NA}=D/\sqrt{D^{2}+4f^{2}}$ . To ensure a consistent image scale across different focal lengths, the actual image height obtained from ray tracing is used as the field parameter. Five equally spaced fields are sampled, corresponding to image heights of 0, 0.4, 0.8, 1.2, and 1.6 mm. A damped least-squares optimization is performed with the root-mean-square (RMS) spot radius as the objective function.

The optimized system layout is shown in Figure 3(a). Both ML1 and ML2 use SiO_2_ substrates, with thicknesses *L*_1_ = 2.8 mm and *L*_2_ = 0.6 mm, respectively. According to the ray tracing results, to avoid vignetting and maintain uniform illumination across a wide field of view, the effective aperture of the quadratic phase surface must cover at least a circular region with radius *ρ*_max_ = 2.1 mm. As shown in Figure 3(b), the back focal length *L*_*b*_ increases linearly with the focal length. Meanwhile, the aperture gradually shifts away from the front surface of ML1 as the focal length increases, though the rate of this displacement slows down. At *f* = 22 mm, the distance *L*_*h*_ reaches a maximum of 6.75 mm. The nonlinear variation of *L*_*h*_ can be realized using a cam mechanism, a technique widely adopted in commercial zoom lenses. The quadratic coefficient *A* in Equation (3) is also obtained through optimization, as shown in Figure 3(c). These optimized values allow for straightforward calculation of the rotation angle *θ* required for ML2 at each focal length, as well as the offset focal length *f*_*offset*_:

(5) $$\begin{array}{c} \begin{array}{c} a=\frac{\left[A\left(f=22\right)-A\left(f=2.2\right)\right]}{\theta_{\max}},\\ \theta \left(f\right)=\frac{\left[A\left(f\right)-A\left(f=22\right)\right]}{a},\\ f_{o\mathrm{ff}set}=\frac{\pi }{\lambda A\left(f=22\right)}, \end{array} \end{array}$$

where *θ*_max_ denotes the maximum rotation angle of ML2. Equation (5) establishes the relationship between the geometrically optimized parameters and the Moiré metalens configuration. A small value of *a* is desirable, as it governs the radial phase gradient at the metalens edge. A larger gradient demands smaller unit cell periods *p*, as given by:

(6) $$\begin{array}{c} p<\frac{1}{2a\rho_{\max}}, \end{array}$$

where *ρ*_max_ denotes the maximum radius of the phase modulation region. By selecting *θ*_max_ = *π*, we obtain *a* = −384.51 mm^−2^, corresponding to a required period *p* < 619.22 nm. The red curve in Figure 3(c) shows the variation of *θ* across different focal lengths. When ML2 is rotated by *θ* *=* 0, 5, 13.3, 30, 80, and 180°, the system achieves focal lengths of *f* = 22, 17.6, 13.2, 8.8, 4.4, and 2.2 mm, respectively.

**Figure 4:**
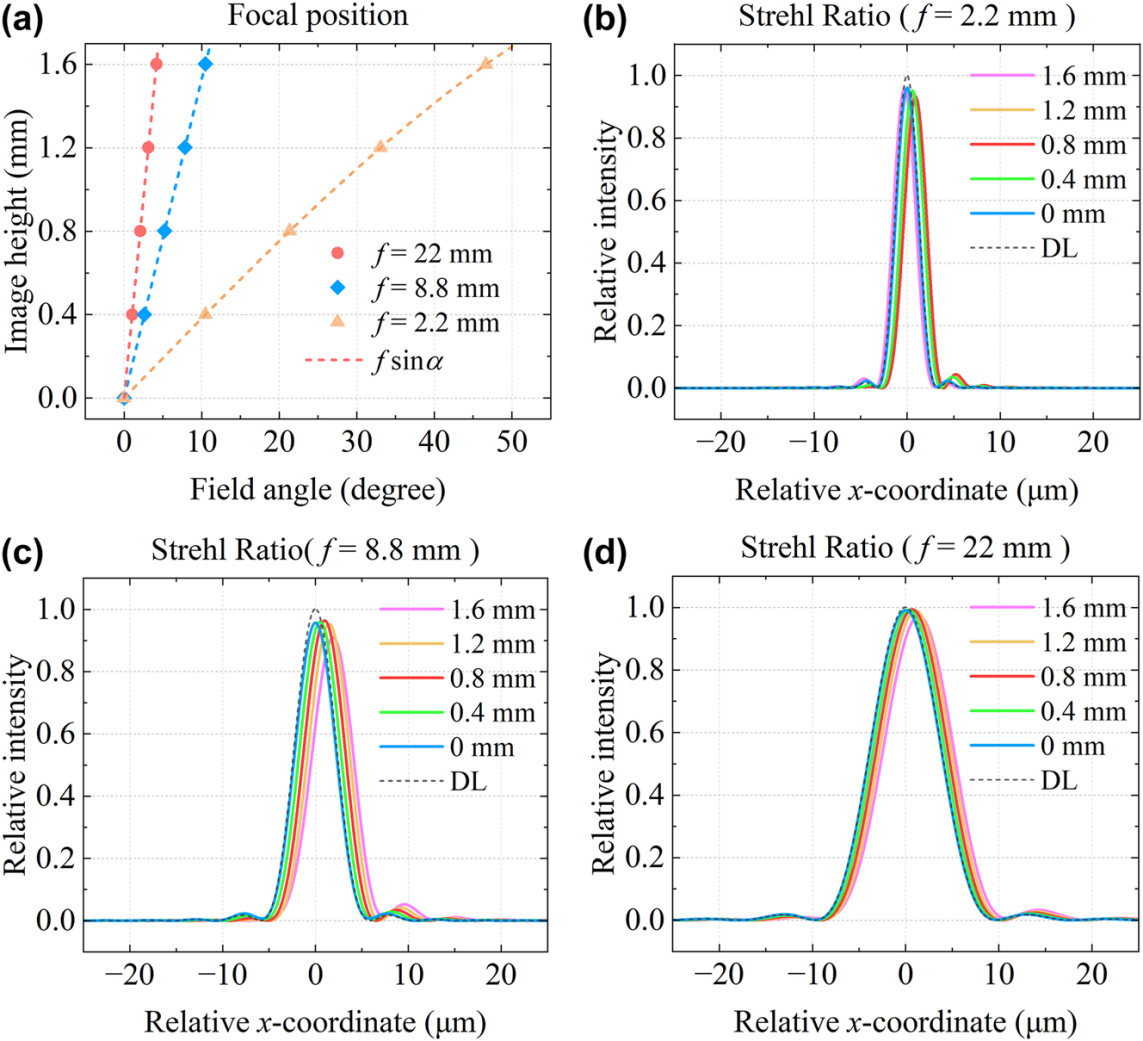
Field angle and Strehl ratio analysis across different focal lengths in the Moiré metalens zoom system. (a) Field angle versus image height at optimized focal lengths of *f* = 2.2, 8.8, and 22 mm. Strehl ratios at various image heights for focal lengths of (b) *f* = 2.2 mm, (c) *f* = 8.8 mm, and (d) *f* = 22 mm, respectively. Five field positions are sampled, corresponding to image heights of 0, 0.4, 0.8, 1.2, and 1.6 mm. DL denotes the diffraction limit.

In wide-angle imaging, the relationship between the field angle and image height is crucial, as it characterizes image distortion. As illustrated in Figure 4(a) and Figure S3(a), the image height is held constant across all focal lengths in our design. At a fixed image height of *h* = 1.6 mm, the corresponding half FOV angles are *α* = 46.64, 21.33, 10.49, 6.97, 5.22, and 4.17° for focal lengths of *f* = 2.2, 4.4, 8.8, 13.2, 17.6, and 22 mm, respectively. During zooming, the total FOV decreases from 93.28 to 8.34°, achieving a zoom ratio of up to 10×. An ideal distortion-free lens satisfies the condition *h* = *f* tan*α*, and distortion is defined as the percentage deviation from this ideal image height. Our Moiré metalens system closely follows the relation *h* = *f*sin*α*, from which the maximum distortion at each focal length can be calculated as 31.26 %, 6.83 %, 1.62 %, 0.68 %, 0.35 %, and 0.21 %, respectively. This introduces barrel distortion, which is particularly noticeable at the shortest focal length. However, for all other focal lengths, the distortion is minimal and visually negligible, as clearly demonstrated in the subsequent imaging results. Distortion is common in conventional wide-angle lenses and does not compromise optical resolution. Furthermore, it can be effectively corrected using standard image processing algorithms [49], [50].

In classical optics, the Strehl ratio is defined as the ratio of the peak on-axis irradiance at the focal point of an actual optical system to that of an ideal diffraction-limited system under identical conditions. It is widely used as a criterion for evaluating the proximity of an imaging system to the diffraction limit. A Strehl ratio above 0.8 is generally accepted as indicative of near-diffraction–limited performance [51], [52]. Figure 4(b) and (c) and Supplementary Figure S2(b) and (c) present the Strehl ratios across different image heights for various focal lengths. The proposed metalens system consistently achieves Strehl ratios exceeding 0.9 across the entire zoom range and full field of view. At both the telephoto end (*f* = 22 mm) and the short-to-mid focal length (*f* = 4.4 mm), the Strehl ratio approaches 1, demonstrating outstanding imaging performance.

**Figure 5:**
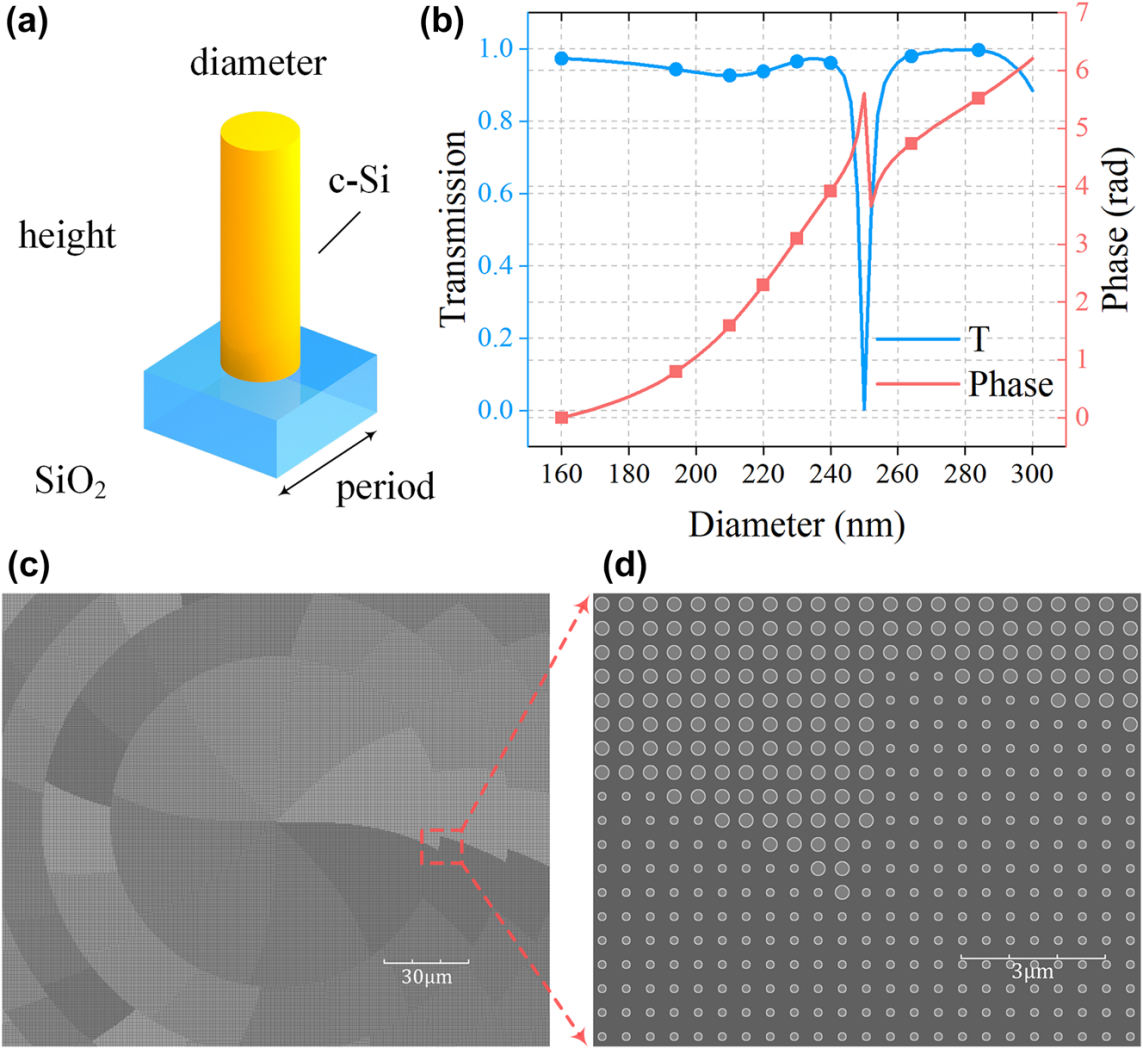
Cylindrical meta-atom design and arrangement in the Moiré metalens zoom system. (a) Schematic of the cylindrical meta-atom. (b) Simulated transmission amplitude and phase of crystalline silicon nanopillars with a height of 800 nm and a period of 500 nm, calculated using commercial FDTD software as a function of diameter. (c, d) Meta-atoms distribution in the central region of ML1’s back surface based on the optimized discretized phase profile.

After obtaining the target phase profiles for the metalenses, a set of meta-atoms capable of covering the full 0–2π phase range must be selected to discretize the design. To maximize optical throughput, polarization-insensitive cylindrical meta-atoms were adopted, as illustrated in Figure 5(a). The nanopillars are made of crystalline silicon [53] and supported by a SiO_2_ substrate [54], both of which are well-established materials compatible with low-cost and scalable nanofabrication processes [55]. To satisfy the constraint set by Equation (3), a square lattice with a subwavelength period of 500 nm was chosen. Using finite-difference time-domain (FDTD) simulations, the transmission amplitude and phase response were characterized for a range of nanopillar heights and diameters, as shown in Figure S3. From this dataset, eight optimized geometries were selected to uniformly cover the 0–2π phase range. As shown in Figure 5(b), all selected meta-atoms have a fixed height of 800 nm, and their diameters vary from 160 nm to 284 nm. These structures achieve an average transmission efficiency exceeding 95 %. Based on the optimized system parameters, the continuous phase profile was quantized into eight discrete levels, and the corresponding meta-atoms were arranged on the metalens surface accordingly to form a circular effective area with a radius of 2.1 mm. Figure 5(c) and (d) depict the meta-atoms distribution in the central region of ML1’s back surface, where the phase gradient gives rise to visible grayscale contrast in the structural pattern.

While geometric optics enables parameter optimization, more rigorous validation is carried out using scalar diffraction theory. In the scalar diffraction simulation model, ML1 and ML2 are treated independently, with their respective phase and transmission profiles discretized using the meta-atoms dataset shown in Figure 5(b). The separation between the two metalenses is set to half of the Talbot distance, i.e., *p* ^2^/*λ*. The simulations employ the band-limited angular spectrum method, which addresses the limitations of conventional angular spectrum propagation in long-distance scenarios [56]. To accommodate the periodic boundary condition intrinsic to the Fast Fourier Transform (FFT), the simulation domain is defined as an 8 × 8 mm^2^ square, with sufficient air margins to suppress artificial edge effects. The sampling interval is matched to the meta-atom period, resulting in a high-resolution grid of 16,000 × 16,000 points.

**Figure 6:**
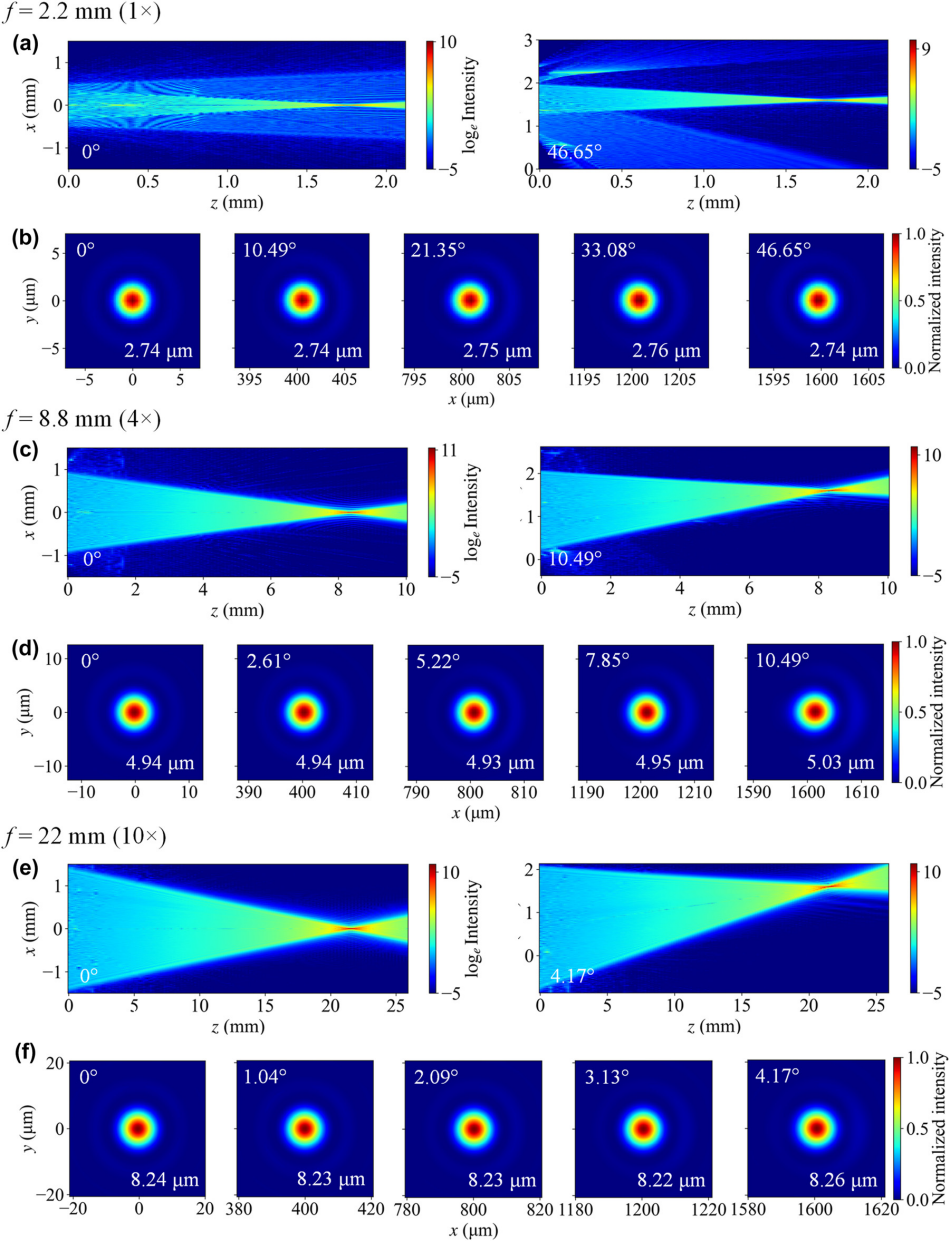
Simulation results using the band-limited angular spectrum method for focal lengths of *f* = 2.2, 8.8, and 22 mm. (a, c, e) Log-scale intensity distributions in the *x–z* (meridional) plane at the minimum and maximum field angles, showing beam propagation from ML2 to the focal plane. (b, d, f) Normalized intensity distributions in the *x–y* focal plane. The top-left and bottom-right corners indicate the field angle and the full width at half maximum (FWHM), respectively. The reported FWHM is the average value along the *x* and *y* directions.

The point spread function (PSF) of the wide FOV zoom imaging system is defined as the normalized intensity distribution at the focal plane produced by the system when illuminated with a plane wave, as shown in Figure 6(b, d, f) and Figure S4(b, d, f). The PSFs exhibit a highly symmetric circular shape across all focal lengths, indicating that spherical aberration has been well suppressed and that off-axis aberrations such as coma and astigmatism have been effectively eliminated. The full width at half maximum (FWHM), averaged over the *x* and *y* directions, is annotated in each figure. According to Equation (4), the theoretical FWHMs under diffraction-limited conditions for *f* = 2.2, 4.4, 8.8, 13.2, 17.6, and 20 mm are calculated to be 2.74, 3.84, 4.93, 6.03, 7.12, and 8.22 μm, respectively. The simulated FWHMs are in excellent agreement with these theoretical values, further confirming that the proposed design achieves near-diffraction–limited performance across the zoom range. Notably, the PSFs remain highly consistent across different field angles, with FWHM variations less than 100 nm, despite all spots being computed at the same focal plane. This demonstrates that field curvature in the zoom system is minimal.

To visualize the beam focusing process, we sampled 200 axial positions along the *z*-axis and plotted the beam propagation from ML2 to the focal plane in Figure 6(a, c, e) and Figure S4(a, c, e). For the short focal lengths (*f* = 2.2 mm and 4.4 mm), a weak diverging beam is observed in addition to the main focused beam, which becomes apparent only after logarithmic intensity scaling. This effect arises from reduced focusing efficiency at large rotation angles of the Moiré lens [30]. As seen in Equation (2), when *θ* = *π*, the first exponential term becomes $\exp\left[i\text{round}\left(a\rho^{2}\right)\pi \right]$ , which simplifies to two-level phase values of 0 or *π*. Insufficient phase levels lead to decreased focusing efficiency. In our design, an 8-level discretization was adopted as a trade-off between fabrication complexity and optical performance [57]. The Moiré metalens experiences two steps of phase quantization: one due to the rotation of ML2 and the other due to the discrete phase levels of meta-atoms. When *θ* ≤ *π*/4, the rotation-induced phase discretization becomes equivalent to 8 levels, and the meta-atoms design becomes the primary limiting factor in phase accuracy. Although some unfocused light is inevitable at short and medium focal lengths, its impact on image quality is negligible. As shown in Figure 6(a), this residual light is highly divergent, and its intensity at the focal plane is below *e* ^−10^ of the peak value. Consequently, its adverse impact can be easily suppressed by slightly increasing the threshold of the CMOS sensor.

**Figure 7:**
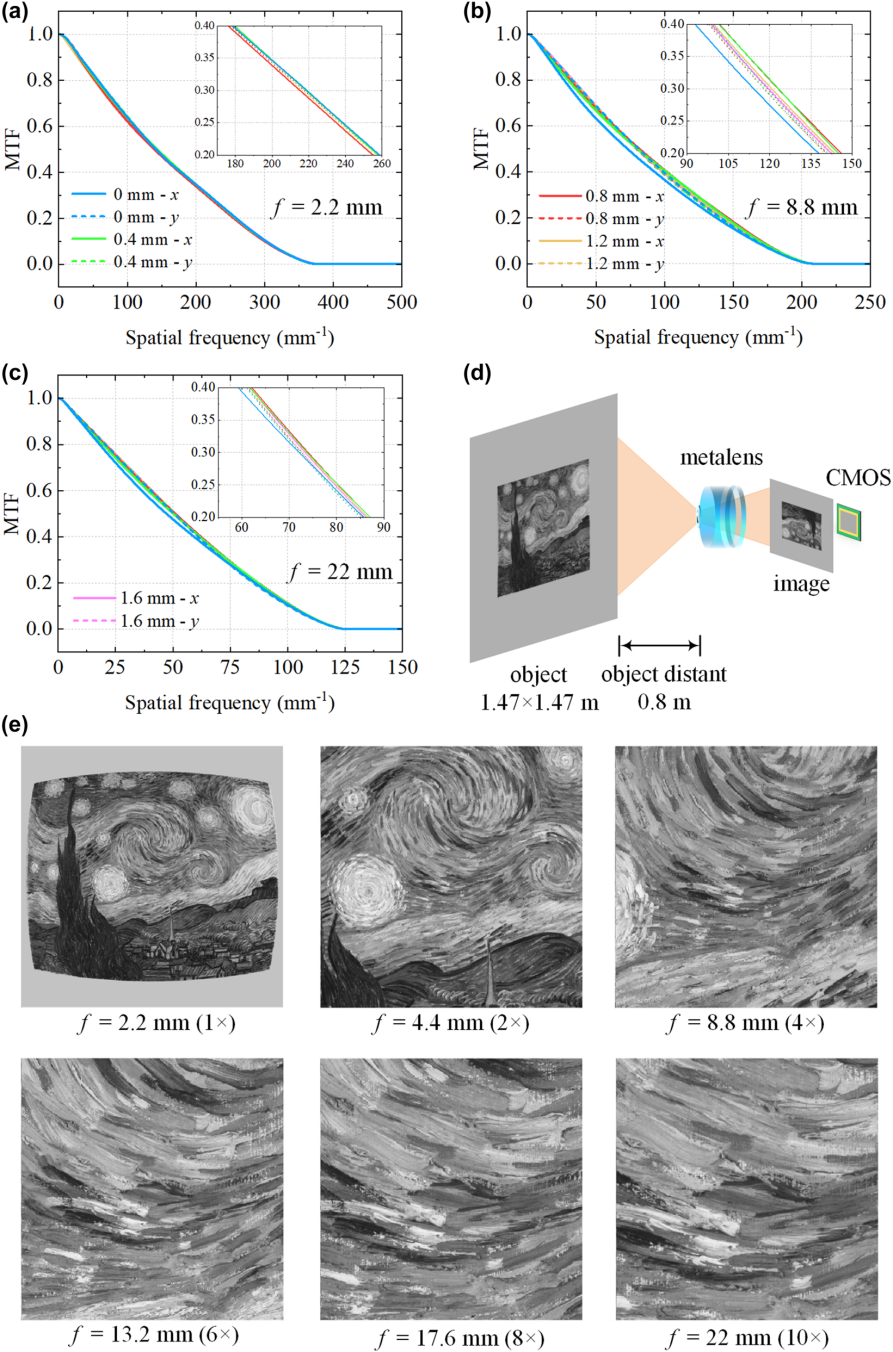
Modulation transfer function (MTF) curves calculated from the PSFs at focal lengths (a) *f* = 2.2 mm, (b) *f* = 8.8 mm, and (c) *f* = 22 mm, respectively. Insets show enlarged views of the MTF curves in the spatial frequency range of 0.2–0.4. Five field angles were considered in the MTF calculation, corresponding to actual image heights of 0, 0.4, 0.8, 1.2, and 1.6 mm. (d) Schematic of the imaging model used for simulating the wide field-of-view Moiré zoom metalens system. (e) Imaging results under different zoom ratios from 1× to 10×.

The modulation transfer function (MTF) describes the contrast performance of an optical system when imaging targets with different spatial resolutions. As the Fourier transform of the PSF, the MTF curves of the zoom metalens system are presented in Figure 7(a)–(c) and Figure S5. The MTF cutoff frequency gradually decreases with increasing focal length, since a longer focal length requires a larger aperture to maintain the same diffraction-limited resolution as a shorter one. One of the key advantages of the variable aperture in our design is its ability to mitigate this degradation in resolution at the telephoto end. In previous studies on Moiré zoom metalens [33], [34], [35], [36], a fixed aperture size was typically used across all focal lengths, which significantly reduces the resolution limit at long focal lengths. Even if the system approaches the diffraction limit, such a configuration may not deliver high imaging resolution. For instance, in our design, if a fixed entrance pupil diameter of 0.88 mm (corresponding to the short focal length) were used at *f* = 22 mm, the f-number would increase to 25, and even an ideal lens would have an MTF cutoff frequency of only 80 mm^−1^. In contrast, our variable aperture expands to 2.93 mm at *f* = 22 mm, increasing the cutoff frequency to 125 mm^−1^. In the design phase, it is generally required that the MTF remain above the empirical threshold of 0.3. At this MTF value, the corresponding spatial frequencies for *f* = 2.2, 4.4, 8.8, 13.2, 17.6, and 20 mm are 215, 152, 113, 95, 82, and 73 mm^−1^, respectively – demonstrating that the system approaches diffraction-limited resolution across the entire zoom range at a wavelength of 1,064 nm. The MTF variation across different field angles is less than 0.1 at the same spatial frequency, indicating high image uniformity over the entire FOV and avoiding the typical degradation in edge image quality. The near-perfect overlap of MTF curves along the *x* and *y* directions further validates the effective suppression of astigmatism in the system.

To further evaluate the imaging performance of the zoom system, we conducted PSF-based image simulations. As shown in Figure 7(d), a grayscale version of Van Gogh’s *The Starry Night* (original size: 92 × 73 cm) is placed on a 1.47 × 1.47 m wall in our modeled scene. The Moiré zoom metalens, positioned 0.8 m from the painting, forms a reduced and inverted image onto a CMOS sensor. We model the imaging process using typical parameters of a near-infrared–enhanced CMOS sensor, with the pixel pitch set to 3.45 μm. The imaging results across a 1–10× zoom range are presented in Figure 7(e). Considering the designed image height of 1.6 mm, we extracted a square region with a 3.2 mm diagonal from the image plane and flipped it for display. At the shortest focal length, the system provides a wide FOV, capturing the entire painting with high fidelity, showing only mild barrel distortion typical of wide-angle lenses. As the focal length increases, the image is progressively magnified. At the telephoto end, the vivid brushwork of the swirling night sky is clearly resolved. High-quality imaging is achieved throughout the entire zoom range, with negligible aberrations observed that could degrade image sharpness.

**Figure 8:**
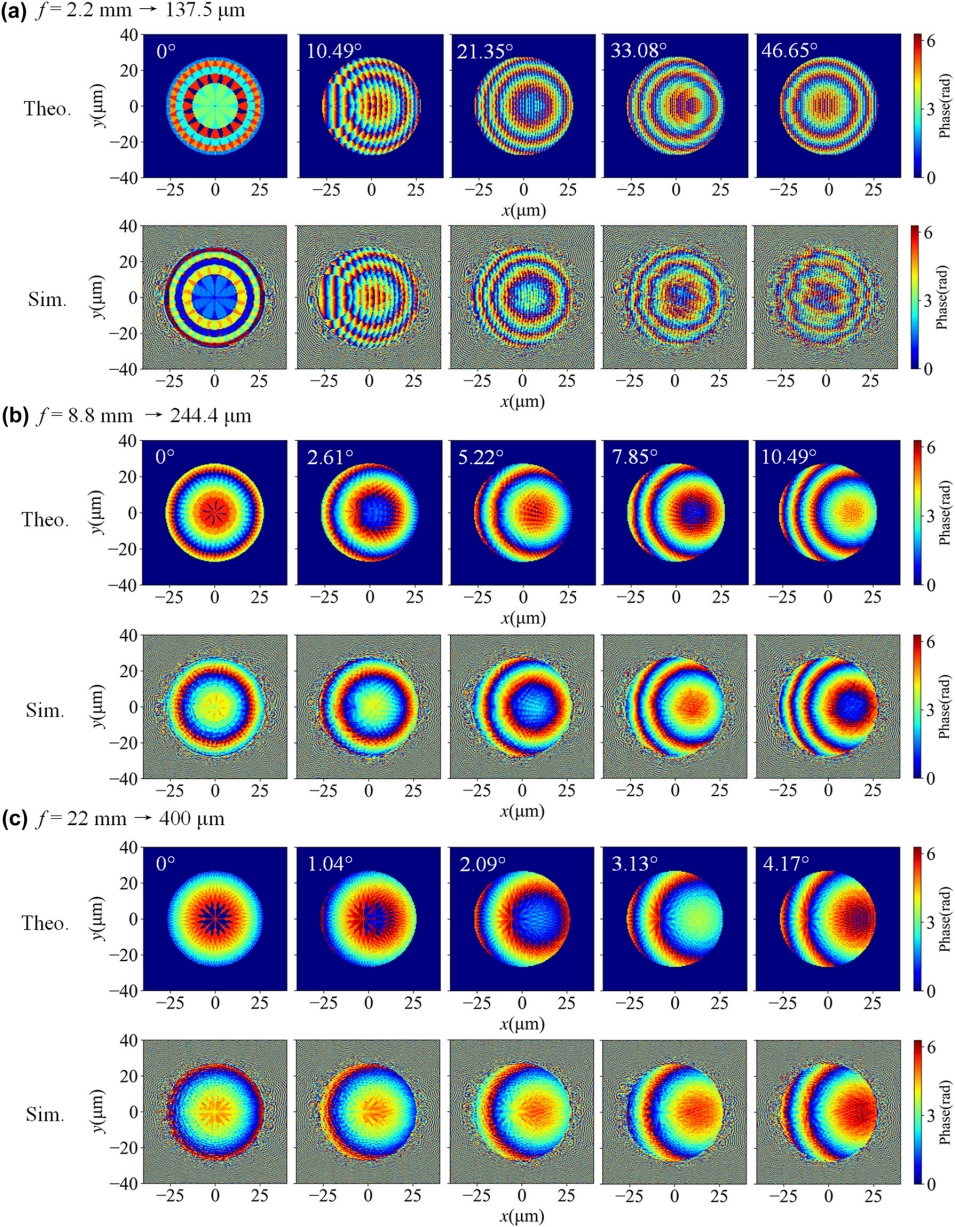
Simulated phase distributions obtained from the FDTD solver of wide FOV Moiré metalens with original focal lengths of (a) *f* = 2.2 mm, (b) *f* = 8.8 mm, and (c) *f* = 22 mm, respectively. The simulations were performed by uniformly scaling down the physical dimensions while keeping the F-number constant, in order to maintain the same diffraction-limited condition. The plots compare the phase profiles recorded at a plane located half a wavelength beyond ML2’s front surface with the corresponding theoretical predictions.

To validate the final design of the wide FOV Moiré zoom metalens, we employed rigorous vector diffraction theory, known for its high accuracy in modeling light–matter interactions at the subwavelength scale. Simulations were performed using a commercial FDTD solver. Due to the substantial computational cost associated with vector diffraction, the metalens structures with focal lengths *f* = 2.2, 8.8, and 22 mm were proportionally downscaled by factors of 16, 36, and 55, respectively, while maintaining a constant f-number. To further reduce computational complexity, simulations were confined to circular regions on ML1 and ML2, corresponding to the projection of the incident beam under each field angle, and each FOV was treated independently [44]. In each simulation model, the diameter of an individual circular region was limited to ∼60 μm, resulting in a total of ∼26,000 meta-atoms across both metalenses surfaces. Figure 8 presents the simulated phase distributions at a plane located half a wavelength behind the front surface of ML2 for the cases of (a) *f* = 2.2 mm, (b) *f* = 8.8 mm, and (c) *f* = 22 mm. The theoretical phase profile Φ_theo_ is given by:

(7) $$\begin{array}{c} \Phi_{theo}=\Phi_{1}+\Phi_{2}\left(\theta \right)+\Phi_{p}\left(\alpha \right), \end{array}$$

where Φ_1_ and Φ_2_ represent the ideal phase distributions imparted by ML1’s back surface and ML2’s front surface as defined in Equation (2), and $\Phi_{p}\left(\alpha \right)$ denotes the phase of a plane wave incident at an angle *α* with respect to the optical axis. After accounting for near-field interactions among the meta-atoms, the combined effect of the two surfaces still closely approximates the ideal phase profile, with only a constant phase offset arising from the sampling location. Notably, under a large field angle of 46.65°, slight deviations from concentric phase contours are observed. These fluctuations originate from the angular-dependent response of individual meta-atoms and result in a marginal reduction in focusing efficiency, without significantly compromising overall performance.

**Figure 9:**
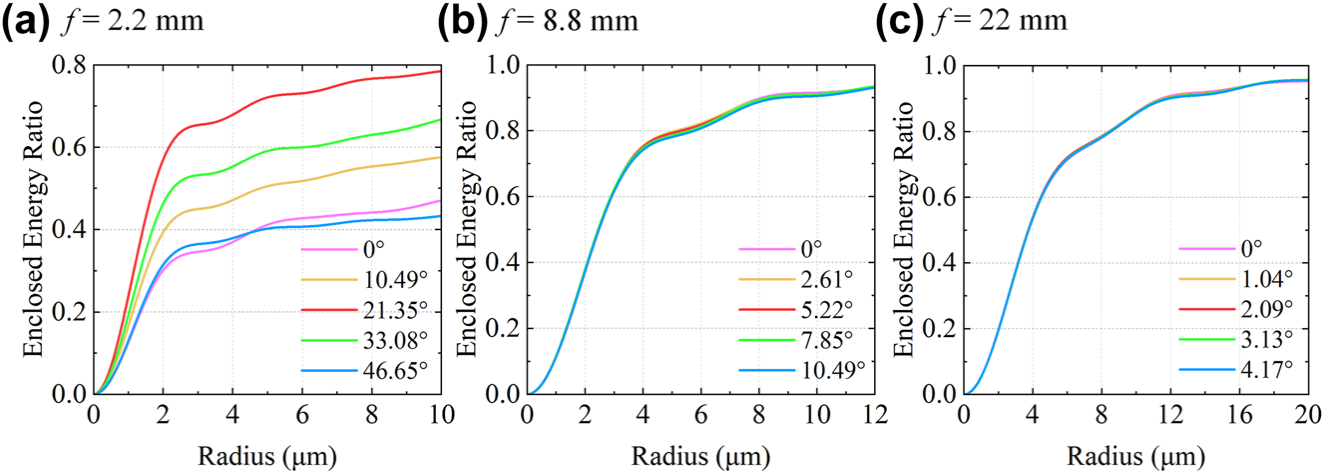
Encircled energy ratios under different FOV angles for scaled-down moiré metalens with original focal lengths of (a) *f* = 2.2 mm, (b) *f* = 8.8 mm, and (c) *f* = 22 mm, respectively.

The electric field components obtained from the FDTD simulations were further propagated using the Rayleigh–Sommerfeld diffraction integral [58] to evaluate the focusing behavior. The resulting optical field distributions in the *x–z* plane are shown in Figure S6, demonstrating well-defined focal spots for all three focal lengths. To quantify the focusing performance, we calculated the encircled energy ratio within a circular area of radius *ρ*_0_ on the focal plane, defined as:

(8) $$\begin{array}{c} \eta \left(\rho_{0}\right)=\frac{\int_{0}^{\rho_{0}}\int_{0}^{2\pi }I\left(\rho ,\varphi \right)\rho d\rho d\varphi }{\int_{0}^{\infty }\int_{0}^{2\pi }I\left(\rho ,\varphi \right)\rho d\rho d\varphi }, \end{array}$$

where $I\left(\rho ,\varphi \right)$ denotes the intensity distribution on the focal plane. The encircled energy within a radius of 1.5×FWHM is used as a metric for focusing efficiency, i.e., $\eta \left(\rho_{0}=3\text{FWHM}/2\right)$ . The computed efficiencies are summarized in Table S1. As shown in Figure 9(a), the encircled energy ratio varies noticeably across different field angles for the short focal length case. This behavior arises primarily from two factors. As previously discussed, the Moiré effect leads to phase quantization into two discrete levels (0 and *π*) at the wide-FOV end, resulting in reduced diffraction efficiency. The energy leakage induced by large rotation angles has a stronger effect at small field angles. At large field angles, the transmission of the meta-atoms inevitably decreases, and their phase response deviates from the target phase, as shown in Figure 8(a). In this regime, the angular sensitivity of the meta-atoms becomes the dominant factor contributing to the decrease in encircled energy ratio. Consequently, the overall focusing efficiency is lower at the wide-FOV end. This issue can be alleviated by adjusting *f*_offset_ to redistribute the rotation angles among different focal lengths. For example, assigning a rotation angle of 0° to the mid focal length and ±90° to the short and long focal lengths provides a more balanced trade-off, which improves the efficiency at short focal length while moderately reducing it at long focal length. Although a reduction is observed at the wide-angle end, the average focusing efficiency still reaches 48.29 %. For the medium and long focal lengths, the focusing efficiencies are more consistent across different field angles, with mean values of 83.91 % and 83.77 %, respectively.

Finally, we performed a tolerance analysis of the interlens separation between ML1 and ML2, as well as the lateral misalignment of the two metalenses along the *x* or *y* direction (see Supplementary Section S3 for details). The results indicate that the short focal length is considerably less sensitive to errors compared with the long focal length. Overall, the imaging performance degradation remains acceptable when the separation is below 4*λ* and the lateral misalignment in a single direction is below 2p. These findings provide practical guidelines for managing fabrication and assembly tolerances in the implementation of the zoom system.

Table 1 compares the performance parameters of this work with those of other similar studies. The commercially available Canon RF lens has a comparable zoom ratio and F-number, but our design offers a wider FOV and a smaller form factor. A 2019 Moiré lens simulation achieved a 30° FOV and approximately 1.5× zoom ratio [40], while our system surpasses this in imaging quality, FOV, and zoom range. A 2022 report on polarization-multiplexed varifocal metalens demonstrated a zoom ratio of 10× with near-diffraction–limited imaging quality, but it only provided two discrete focal lengths and lacked continuous zoom functionality, with a FOV of only 40° at the wide angle [29]. Most recently reported metalens either offer a wide FOV or zoom functionality, but rarely both simultaneously [23], [28], [42]. Among reported metalens-based systems that offer both zoom functionality and a wide FOV, our design demonstrates the broadest angular coverage and the largest continuous zoom ratio to date. Although experimental implementation is beyond the scope of this work due to our equipment and fabrication limitations, the super-wide FOV zoom moiré metalens has been thoroughly validated through multiple simulation methods and theoretical models. Both its structural dimensions and material choices are compatible with current fabrication technologies [59], [60], [61].

**Table 1:** Performance comparison between this work and previously reported zoom or wide-field imaging systems.

Reference	This work	Canon RF	2019 [40]	2022 [29]	2023 [28]	2020 [42]	2024 [23]
Method	Moiré	Tradition	Moiré	Polarization	PCM	Fixed-focus	Axial
Wavelength	1,064 nm	Visible	810 nm	5.2 μm	1,550 nm	850 nm	940 nm
Focal length	2.2–22 mm	24–240 mm	9.87–15.21 μm	1.1 and 10.8 mm	41 and 123 μm	3.36 mm	1.4–7.5 mm
F-number	2.5–7.5	4–6.3	0.35–0.54	1.4 and 6.8	1.2 and 1.8	2.5	1.1–5.9
Field of view	93.28°–8.34°	84°–10.33°	30°	40° and 4°	0°	80°	0°
Diffraction limit	True	False	False	True	False	False	False
Size	4.2 × 32 mm	80.4 × 122.5 mm	28 × -μm	4 × 4.82 mm	70 × -μm	2 × -mm	1.28 × -mm

## 4 Conclusions

In this work, we present a novel wide FOV zoomable metalens system, along with a comprehensive multi-theory design and validation framework. The design consists of two Moiré-based metalenses capable of relative rotational tuning and a variable aperture. This configuration enables continuous zooming with a maximum zoom ratio of 10×. It effectively integrates wide-angle, mid-focal, and telephoto functionalities within the near-infrared regime, achieving a full FOV ranging from 8.34° to 93.28°. The inclusion of a variable aperture not only suppresses aberrations but also enhances the cutoff spatial frequency of the MTF at the telephoto end from 80 mm^−1^ to 125 mm^−1^. The optimized system maintains near-diffraction–limited imaging resolution across the full range of focal lengths and field angles, with Strehl ratios exceeding 0.9. The overall system parameters are comparable to those of commercial zoom lenses, while offering a more compact and lightweight solution. This design holds great potential for applications where weight and volume are critical constraints, such as in drones and miniature robotic systems. The integrated design methodology, incorporating geometric optics, scalar diffraction, and full-vector electromagnetic theories, provides a powerful and generalizable framework for the development of next-generation metalens imaging systems.

## Supplementary Material

Supplementary Material Details
