# Supplementary material for: A 10× continuously zoomable metalens system with super-wide field of view and near-diffraction–limited resolution
Source: Nanophotonics. 2025 Nov 24;14(27):5251–65. doi: 10.1515/nanoph-2025-0399 (PMC12717904; doi:10.1515/nanoph-2025-0399)
Supplement: Supplementary file 1 — Supplementary Material Details [file j_nanoph-2025-0399_suppl_001.pdf]

Supplementary Material

A 10× Continuously Zoomable Metalens System with Super-Wide Field of View and Near-Diffraction-Limited Resolution

Wangzhe Zhou[1], Shaoqi Li[1], Yiyi Li[1], Zongyuan Chen[1], ManYuan[1], Fen Zhao[2], YutaiChen[1], Huan Chen[1], Zhaojian Zhang[1], Jiagui Wu[3]*, Junbo Yang[1]*

[1]*College of Science, National University of Defense Technology, Changsha 410073, China*
[2]*School of Artificial Intelligence, Chongqing University of Technology, Chongqing 401135, China*
[3]*School of Physical Science and Technology, Southwest University, Chongqing 400715, China*
*mgh@swu.edu.cn
*yangjunbo@nudt.edu.cn

S1. Moiré lens theory

In the polar coordinate system (ρ, φ), the quadratic phase transmission function of a lens with focal length f can be expressed as:

$$T_{\text{quad}} = \exp\left(-\frac{i\pi\rho^2}{\lambda f}\right). \tag{S1}$$

This function can be interpreted as the product of the transmission functions of two Moiré surfaces:

$$T_{\text{quad}} = \exp(ia\rho^2\varphi)\exp[-ia\rho^2(\varphi-\theta)] = T_1T_2 = \exp(ia\theta\rho^2), \tag{S2}$$

where θ denotes the counterclockwise rotation angle of the second metalens (ML2). The constant a is defined as:

$$a = -\frac{\pi}{\lambda f\theta}. \tag{S3}$$

Considering the practical implementation of the Moiré metalens, which adopts a circular configuration with inherent 2π rotational symmetry, it is important to account for the fact that the function $\exp[-ia\rho^2(\varphi-\theta)]$ is not 2π-periodic. Therefore, correction must be applied to regions outside the principal period:

$$T_2 = \begin{cases} \exp[-ia\rho^2(\varphi-\theta)] & \theta-\pi \le \varphi < \pi \\ \exp[-ia\rho^2(\rho-\theta+2\pi)] & -\pi \le \varphi < \theta-\pi \end{cases} \quad (0 \le \theta < \pi)\ , \tag{S4a}$$

$$T_2 = \begin{cases} \exp[-ia\rho^2(\varphi-\theta)] & -\pi \le \varphi < \theta+\pi \\ \exp[-ia\rho^2(\varphi-\theta-2\pi)] & \theta+\pi \le \varphi < \pi \end{cases} \quad (-\pi \le \theta < 0). \tag{S4b}$$

Accordingly, the combined transmission function of the two Moiré surfaces is revised as:

$$T_{\text{total}} = \begin{cases} \exp(-ia\rho^2\theta) & \theta-\pi \le \varphi < \pi \\ \exp[-ia\rho^2(\theta-2\pi)] & -\pi \le \varphi < \theta-\pi \end{cases} \quad (0 \le \theta < \pi), \tag{S5a}$$

$$T_{\text{total}} = \begin{cases} \exp(-ia\rho^2\theta) & -\pi \le \varphi < \theta+\pi \\ \exp[-ia\rho^2(\theta+2\pi)] & \theta+\pi \le \varphi < \pi \end{cases} \quad (-\pi \le \theta < 0). \tag{S5b}$$

As evident from Equation (S5), the phase profile comprises not only the desired quadratic phase term but also an angular sector exhibiting a focal length mismatch, introducing phase discontinuity. This issue can be effectively resolved by incorporating a rounding operation:

$$T_1 = \exp[i\text{round}(a\rho^2)\varphi], \tag{S6a}$$

$$T_2 = \exp[-i\text{round}(a\rho^2)(\varphi-\theta)]. \tag{S6b}$$

It is straightforward to verify that $\exp[-i\text{round}(a\rho^2)\varphi] = \exp[-i\text{round}(a\rho^2)(\varphi+2\pi)]$, thereby ensuring 2π-periodicity. The resulting cascaded transmission function becomes:

$$T_{\text{tolal}} = \exp[i\text{round}(a\rho^2)\theta]. \tag{S7}$$

To assign a specific focal length f_{offset} to the case where the rotation angle $\theta = 0$, it is sufficient to multiply Equation (S7) by an initial quadratic phase term: $\exp(-i\pi\rho^2/f_{\text{offset}}\lambda)$. To maintain

the symmetry between the two metalens and preserve the cascaded design architecture, this global phase can be evenly distributed across the two layers. Accordingly, both transmission functions T_1 and T_2 are multiplied by a common prefactor: $\exp(-i\pi\rho^2/2f_{\text{offset}}\lambda)$.

To ensure the phase difference between adjacent meta-atoms does not exceed π, the phase gradients must satisfy the following conditions:

$$\frac{\mathrm{d}\Phi}{\mathrm{d}\rho} < \frac{\pi}{p}, \tag{S9a}$$

$$\frac{\mathrm{d}\Phi}{\rho\mathrm{d}\varphi} < \frac{\pi}{p}, \tag{S9b}$$

where p is the minimum unit cell pitch. Using the quadratic phase expression $\Phi = a\rho^2\varphi$, and considering $\varphi \in [-\pi, \pi)$, one obtains the constraint on the periodicity:

$$p < \frac{1}{2a\rho_{\max}}. \tag{S10}$$

Figure S1 presents the phase distributions of the ML2's front surface for counterclockwise rotation angles ranging from 5° to 80°, along with the corresponding total phase profiles. These results clearly demonstrate how the rotation angle θ modulates the total phase. The total phase profile is not continuous due to the combined effects of the $\mathrm{round}(\cdot)$ function and the rotation angle θ. The discrete phase steps—defined as the differences between adjacent quantized levels—are directly proportional to θ. As θ decreases, the phase distribution becomes smoother; conversely, larger θ values lead to more pronounced discretization, resulting in reduced focusing efficiency. It should be noted that the plotted region corresponds to a central area of 0.25×0.25 mm, whereas the actual effective diameter of the metalens is 4.2 mm.

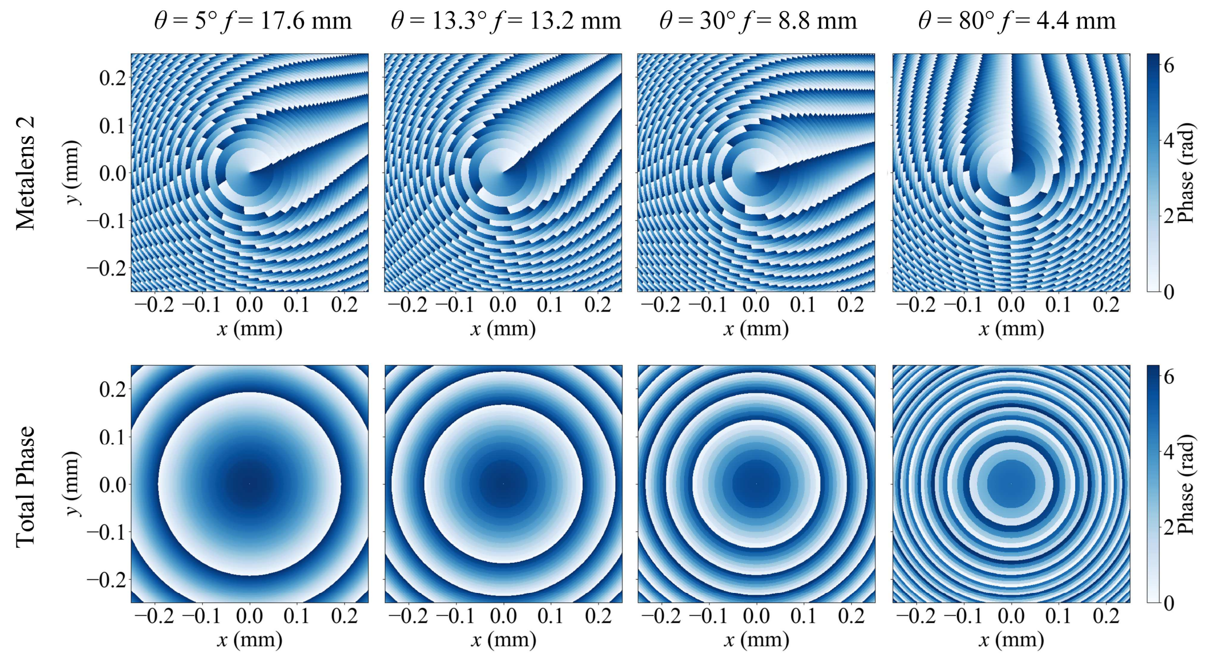

Figure S1. Central-region phase distributions of ML2's front surface, corresponding to focal lengths of 17.6 mm, 13.2 mm, 8.8 mm, and 4.4 mm. The total phase profiles are formed by the superposition of ML2's front surface, rotated by different relative angles θ, with a fixed ML1's back surface. Only a 0.25×0.25 mm region at the center of the metalens is shown for clarity.

S2. Supplementary Design and Validation Data

Before selecting the appropriate meta-atoms, we performed a parametric scan of their geometric parameters. The scanning range for the cylinder diameter was 100–400 nm, and the height was varied between 700 and 900 nm. Figure S4 shows the corresponding transmission and phase profiles of the selected meta-atom structures, obtained using FDTD simulations. These results were used to identify meta-atom designs that not only exhibited the highest transmission efficiency but also satisfied the phase requirements of 0 to 2π.

The calculations using geometrical optics and scalar diffraction considered six different focal lengths: f = 2.2, 4.4, 8.8, 13.8, 17.6, and 22 mm. Due to space constraints, only three focal lengths, f = 2.2, 8.8, and 22 mm, representing wide-angle, mid-focal, and telephoto, are presented in the main text. The remaining focal lengths are provided in Figure S2, S4, S5. The results further validate that the designed metalens system achieves near-diffraction-limited imaging performance across the entire focal range.

Figure S3 presents the phase and transmittance spectra of silicon nanopillars obtained by finite-difference time-domain (FDTD) simulations, with pillar heights ranging from 700 to 900 nm and diameters from 100 to 400 nm. From this dataset, we selected eight optimized geometries that exhibit an average transmittance exceeding 95%, while providing a uniform coverage of the entire 0–2π phase range.

The vectorial light field's x-component, emitted from the front surface of ML2, was extracted and used as the input complex amplitude for the Rayleigh-Sommerfeld diffraction integral algorithm [1], allowing the calculation of the light field distribution in the x–z plane as shown in Figure S6. This also yields the light field distribution in the x–y plane at the focal point, i.e., the PSF. For FDTD simulations, a grid spacing of 30 nm was used, and the same grid density was applied in the Rayleigh-Sommerfeld diffraction calculation.

The focusing efficiency is defined as the ratio of the energy enclosed within a circle of diameter three times the full-width at half-maximum (FWHM) at the focal plane to the total energy. The focusing efficiency was computed for a geometrically scaled model, and Table S1 summarizes the efficiencies η for the original focal lengths f = 2.2, 8.8, and 22 mm. The inherent characteristics of the Moiré lens lead to a decrease in phase sampling as the rotation angle increases, which corresponds to a reduction in focusing efficiency for shorter focal lengths. The average focusing efficiency for the short focal length is approximately 50%, while the mid and long focal lengths exhibit efficiencies greater than 80%.

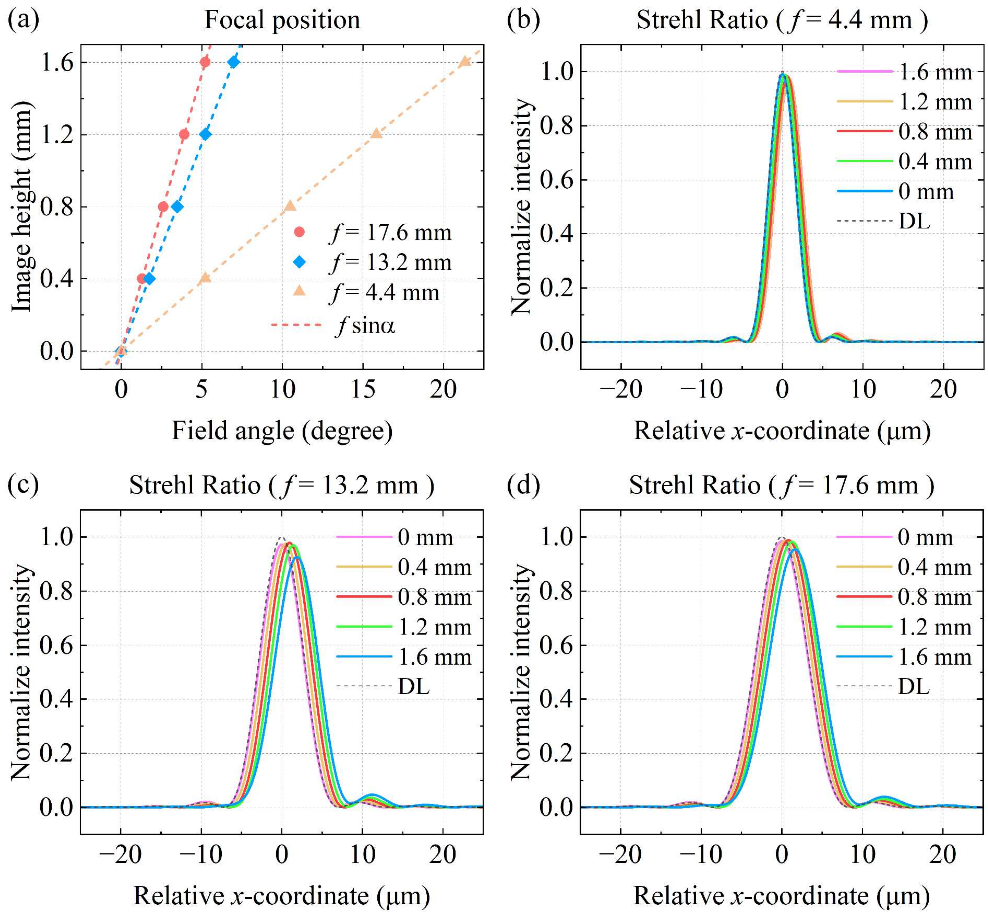

Figure S2. (a) Field angle versus image height at optimized focal lengths of $f = 4.4$, 13.8, and 17.6 mm. Strehl ratios at various image heights for focal lengths of (b) $f = 4.4$ mm, (c) $f =$ 13.8 mm, and (d) $f = 17.6$ mm, respectively. Five field positions are sampled, corresponding to image heights of 0, 0.4, 0.8, 1.2, and 1.6 mm. DL denotes the diffraction limit.

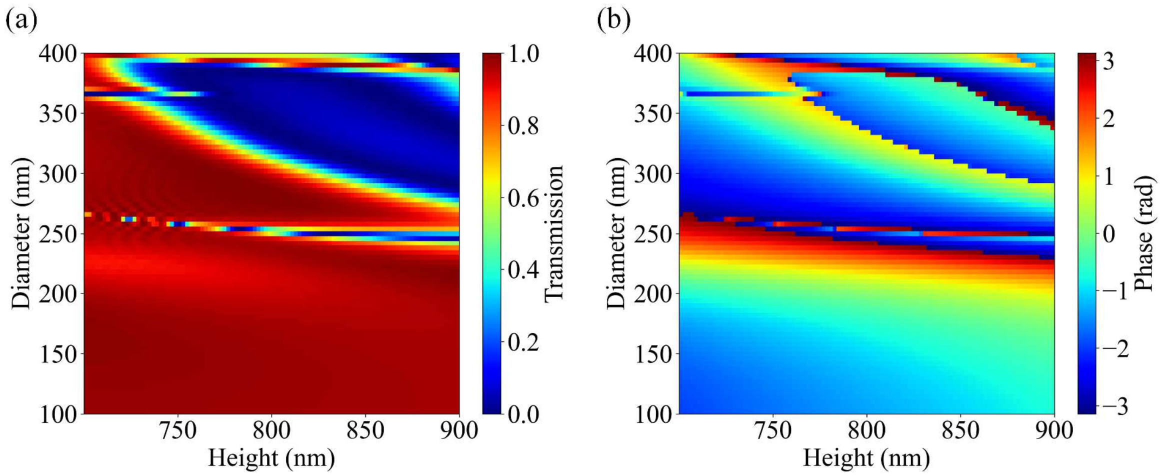

Figure S3. Transmission (a) and phase (b) of meta-atoms with different diameters and heights.

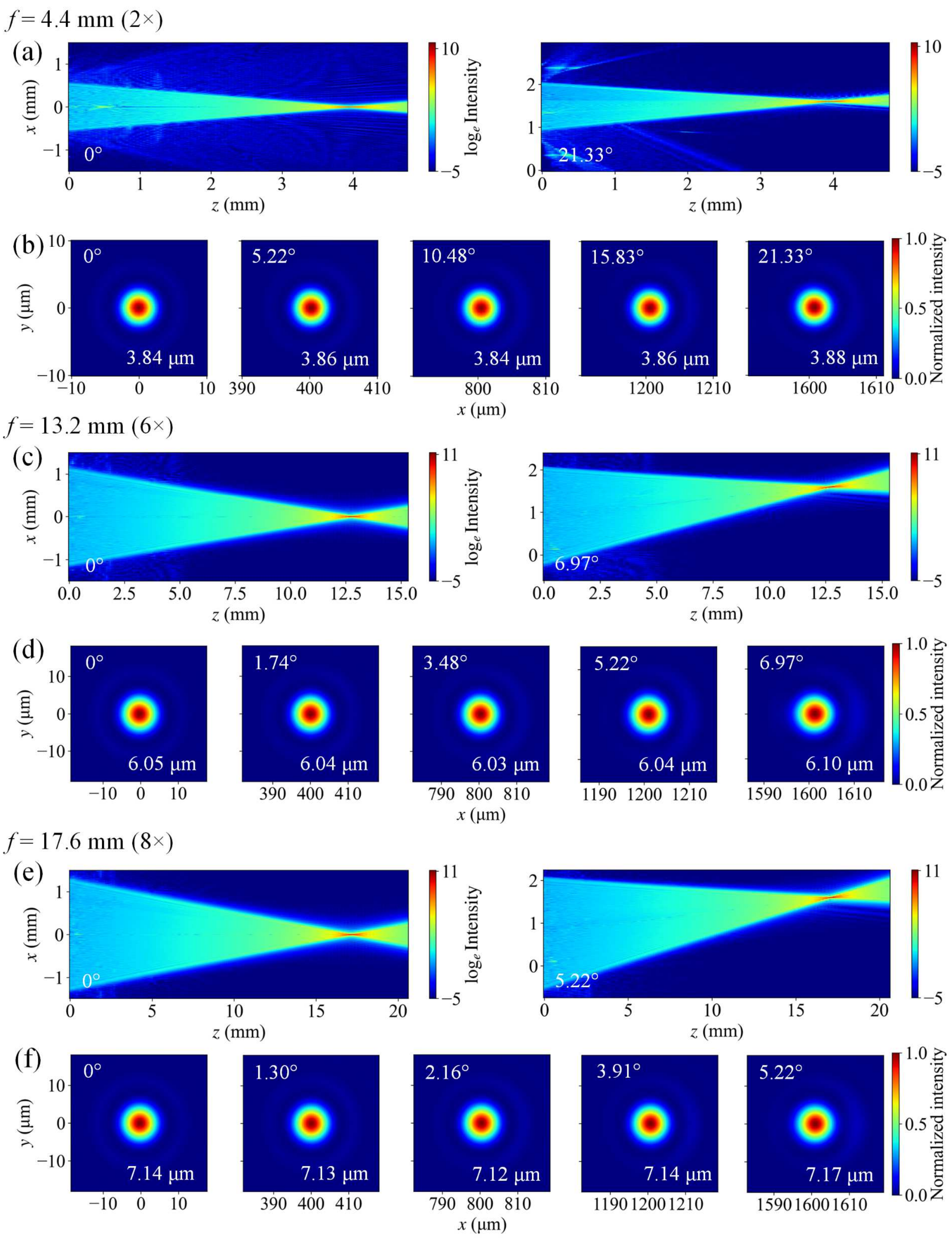

Figure S4. Simulation results using the band-limited angular spectrum method for focal lengths of $f = 4.4$, 13.2 and 17.6 mm. (a, c, e) Log-scale intensity distributions in the x–z (meridional) plane at the minimum and maximum field angles, showing beam propagation from metasurface 2 to the focal plane. (b, d, f) Normalized intensity distributions in the x–y focal plane. The top-left and bottom-right corners indicate the field angle and the full width at half maximum (FWHM), respectively. The reported FWHM is the average value along the x and y directions.

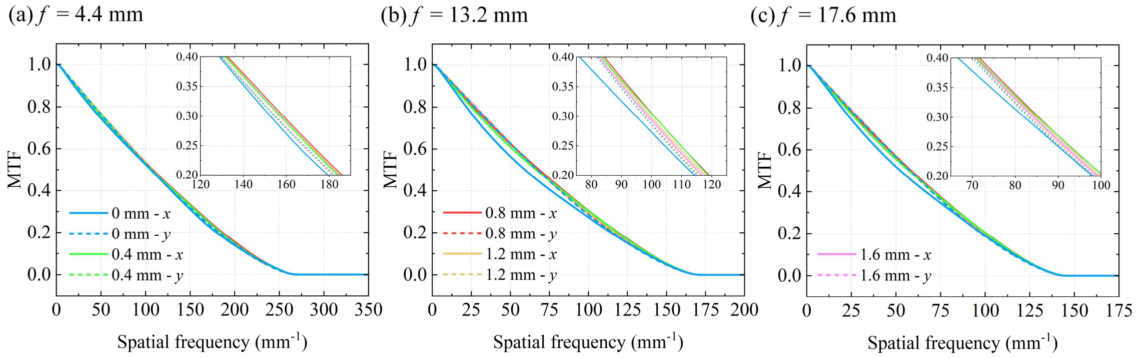

Figure S5. Modulation transfer function (MTF) curves calculated from the PSFs at focal lengths (a) f = 4.4 mm, (b) f = 13.2 mm, and (c) f = 17.6 mm, respectively. Insets show enlarged views of the MTF curves in the spatial frequency range of 0.2–0.4. Five field angles were considered in the MTF calculation, corresponding to actual image heights of 0, 0.4, 0.8, 1.2, and 1.6 mm.

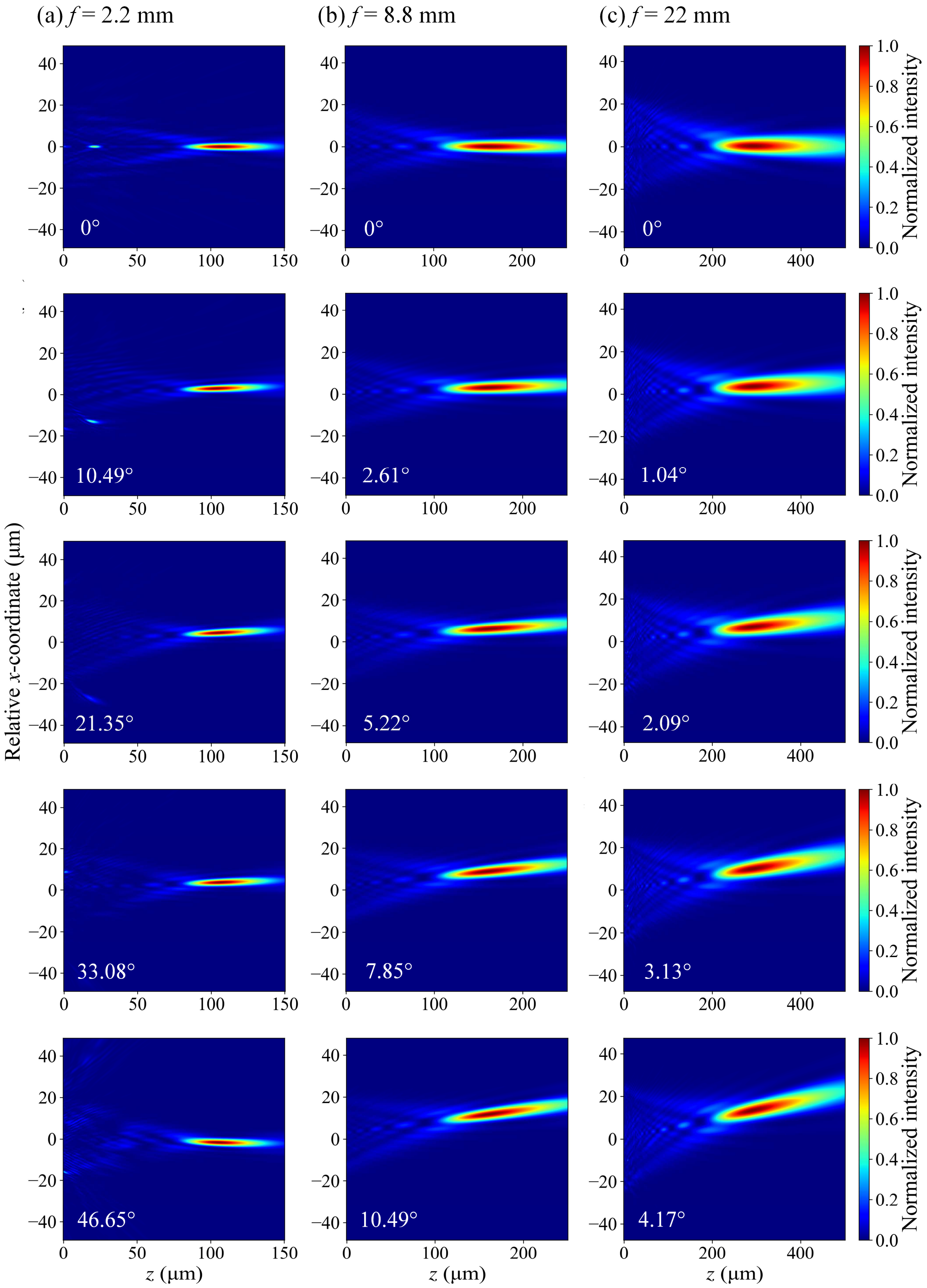

Figure S6. Simulated x–z plane intensity distributions of the Moiré metalens system after proportional downscaling, corresponding to the original focal lengths of (a) $f = 2.2$ mm, (b) $f = 8.8$ mm, and (c) $f = 22$ mm. The simulations were performed using Rayleigh–Sommerfeld diffraction, with the input complex field extracted from the FDTD-calculated output of ML2's front surface.

Table S1. Focusing efficiency η of each focal length after proportional downscaling, evaluated under different field angles α.

f = 2.2 mm		f = 8.8 mm		f = 22 mm	
α (degree)	η (%)	α (degree)	η (%)	α (degree)	η (%)
0	36.8	0	84.83	0	83.88
10.49	44.68	2.61	84.73	1.04	83.68
21.35	67.66	5.22	84.03	2.09	83.95
33.08	55.25	7.85	83.33	3.13	83.83
46.65	38.07	10.49	83.02	4.17	83.52
Avg.	48.492	Avg.	83.988	Avg.	83.772

S3. Tolerance analysis

Using the band-limited angular spectrum method, we investigated the tolerance sensitivity of key parameters in the wide FOV Moiré zoom metalens system. Specifically, we simulated the effects of the separation between ML1 and ML2, as well as the lateral misalignment of the two metalenses along the x or y direction, on the imaging performance of the system. The following sections present the results in detail.

S3.1. Inter-Lens Separation

The separation between the two metalenses was set to 2, 4, 8, 16, and 32 times the wavelength (λ). Under the condition that the back focal length of the original design remained unchanged, the normalized intensity distributions at the focal plane (x–y plane) were calculated, as shown in Figure S7(a) and (b). Two representative focal lengths, a short focal length (f = 2 mm) and a long focal length (f = 22 mm), were selected for analysis. In addition, different field conditions were considered by examining the cases with image heights of 0, 0.8, and 1.6 mm. For the short focal length, these image heights correspond to incident plane-wave angles of 0°, 21.35°, and 46.65°, respectively, while for the long focal length they correspond to 0°, 2.09°, and 4.17°. The sensitivity of the short focal length to separation variations is significantly weaker than that of the long focal length. At f = 2 mm, noticeable distortion of the focal spot appears only when the separation increases to 16λ, and even then only for the on-axis field. This phenomenon is quantitatively described by the full width at half maximum (FWHM), as shown in Figure S8(a). When the separation reaches 16λ, the on-axis field's FWHM in the y direction increases to 3.1 μm. When the separation is increased to 32λ, the FWHM increases in both x and y directions to 2.98 μm and 3.13 μm, respectively. Nevertheless, the short-focus FWHM remains close to the diffraction limit (2.74 μm). Moreover, Figure S7(a) shows that increasing the separation leads to a slight displacement of the focal spot center toward the positive x and y directions, with stronger effects at larger field angles. At a separation of 32λ, the maximum displacement in the y direction is 4.5 μm, occurring at an image height of 1.6 mm (incident angle 46.65°). The maximum displacement in the x direction is 1.5 μm, occurring at an image height of 0.8 mm (incident angle 21.35°). Such center shifts do not directly reduce the lens resolution but introduce image distortion. Fortunately, the maximum shifts are under 5 μm, equivalent to only about 1–2 sensor pixels, and thus are negligible for most application scenarios.

The long-focus case is substantially more sensitive to separation, as shown in Figure S7(b). For f = 22 mm, a separation of 4λ already causes the focal spot to deviate slightly from circular symmetry. When the separation is increased to 16λ, the focal spots at all three fields are severely distorted: the main lobe enlarges markedly and significant background noise appears. At a

separation of 32λ the spot is elongated in the y direction. The FWHMs at image heights of 0, 0.8 and 1.6 mm reach 24.52, 23.06 and 23.45 μm, respectively (Figure S8(b)), which is approximately three times the diffraction limit (8.22 μm).

To more accurately characterize the impact of separation on imaging resolution, we further calculated the modulation transfer function (MTF) under different separations, as shown in Figure S9 (a) and (b). For the short focal length, the MTF remains close to the diffraction limit across all separations. Using the spatial frequency at MTF = 0.3 as the criterion (all spatial frequencies mentioned below are defined at MTF = 0.3), the maximum deviation from the diffraction limit occurs at the on-axis field with a separation of 32λ, where the spatial frequencies in the x and y directions decrease to approximately 180 mm^{-1} and 170 mm^{-1}, respectively.

In the long focal length case, only at a separation of 2λ does the MTF remain close to the diffraction limit. Since the effects of separation are similar across different fields of view, we take the on-axis field as a representative example. At a separation of 4λ, the spatial frequency in the y direction is nearly unaffected, while that in the x direction decreases to 54 mm^{-1}. With further increases in separation, because the elongation of the focal spot in the y direction becomes more pronounced than in the x direction, the spatial frequencies at a separation of 32λ fall to 24 mm^{-1} and 10 mm^{-1} in the x and y directions, respectively.

In summary, when compared with the results under the ideal separation shown in Figure 6(a) and 6(c), the imaging quality can be considered to remain essentially unaffected for separations below 4λ. In currently fabricated Moiré metalenses, an achievable separation of at least 3.6 μm (after accounting for the meta-atom height) has already been demonstrated [2], indicating that the wide FOV Moiré zoom system studied here can be feasibly realized under existing fabrication conditions.

S3.2. Lateral Misalignment

Lateral misalignment between the two metalenses, i.e., misalignment perpendicular to the optical axis (z direction), is also a critical factor affecting imaging quality. We decomposed this effect into misalignments along the x and y directions and simulated them separately. Considering again two focal lengths and three field positions, the misalignments along the x and y axes were set to 2, 4, 8, and 16 periods (p), and the corresponding MTF results are shown in Figure S10.

For the short focal length case (f = 2 mm), both x and y direction misalignments primarily affect the on-axis field (image height = 0 mm), while the image heights of 0.8 mm and 1.6 mm remain close to the diffraction limit. When the misalignment exceeds 8p, the MTF in the

corresponding direction for the on-axis field drops significantly. However, for misalignments of $2p$ and $4p$, the spatial frequency at MTF = 0.3 always remains above 150 mm^{-1}.

The long focal length (f = 22 mm) exhibits a much stronger sensitivity to misalignment, particularly along the y direction. In contrast to the short-focus case, at f = 22 mm the response to misalignment is comparable among the three fields. With an x-direction misalignment of $2p$, the spatial frequency decreases to as low as 36 mm^{-1}. A misalignment of $4p$ still ensures that the spatial frequency stays above 30 mm^{-1} for all fields.

These simulations demonstrate that lateral misalignment has a non-negligible impact on Moiré metalens performance. Therefore, in practical fabrication it is essential to strictly control the processing and assembly tolerances of the two metalenses in the lateral (x–y) directions. At present, most tolerance studies on Moiré metalens have focused on inter-lens separation, and algorithms have been developed to enhance tolerance to separation errors [3]. In contrast, investigations on the effect of lateral misalignment remain scarce. Our simulations may thus provide valuable insights to guide future research in this direction.

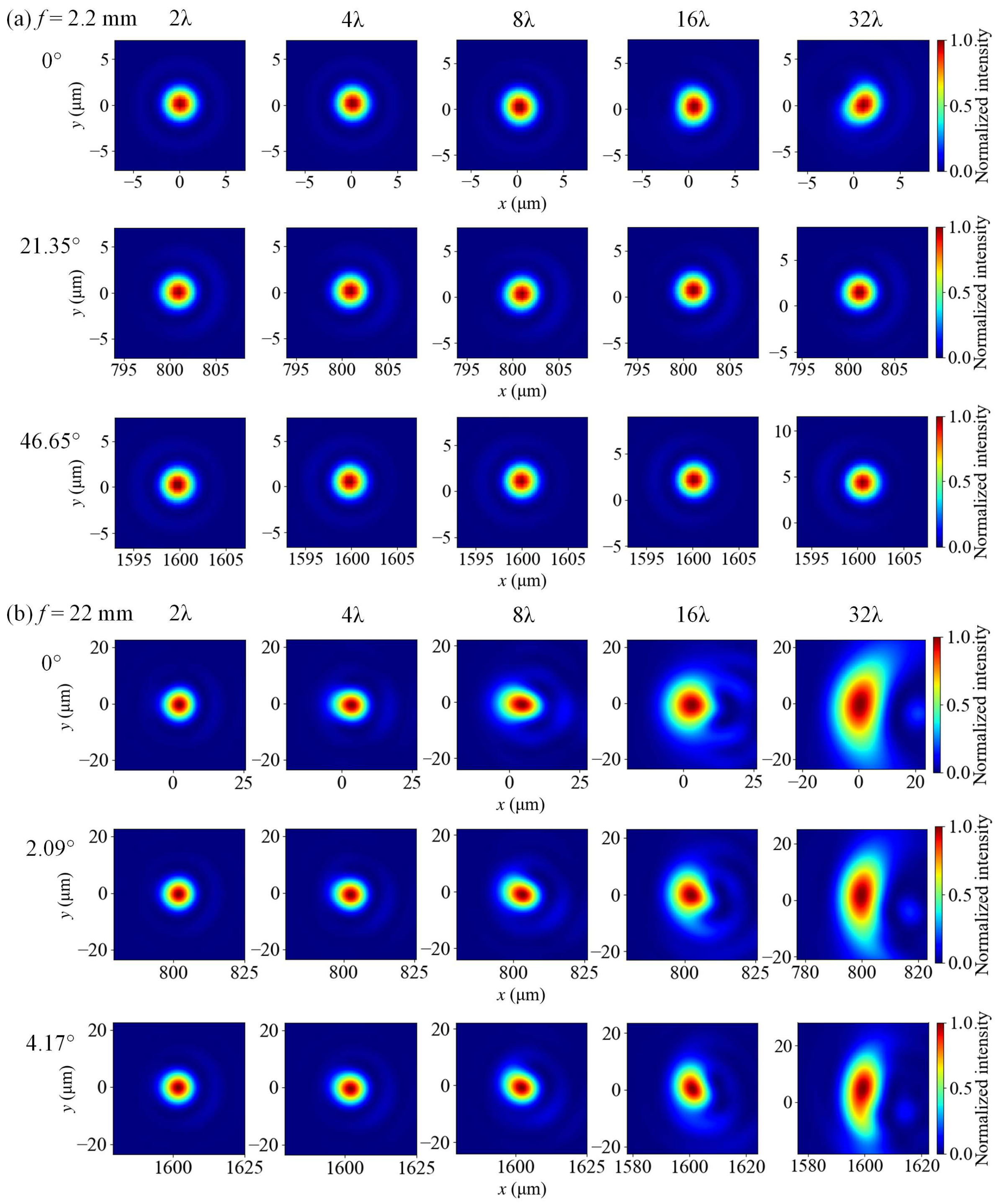

Figure S7. Intensity distributions in the *x*–*y* focal plane when the separation between the two metalenses is set to 2, 4, 8, 16, and 32 times the wavelength (λ). The simulations are performed using the band-limited angular spectrum method, considering focal lengths of (a) f = 2 mm and (b) f = 22 mm. Image heights of 0, 0.8, and 1.6 mm are calculated, and the corresponding field angles are indicated in the figure.

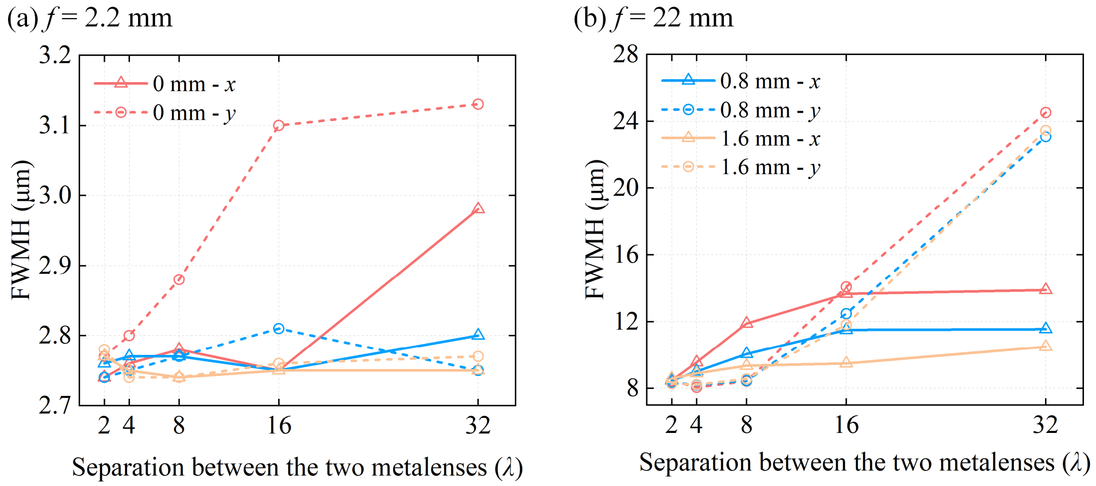

Figure S8. Variation of the focal full width at half maximum (FWHM) with the separation between the two metalenses at focal lengths of (a) $f = 2.2$ mm and (b) $f = 22$ mm. For image heights of 0, 0.8, and 1.6 mm, the FWHM values are calculated separately along the x and y directions.

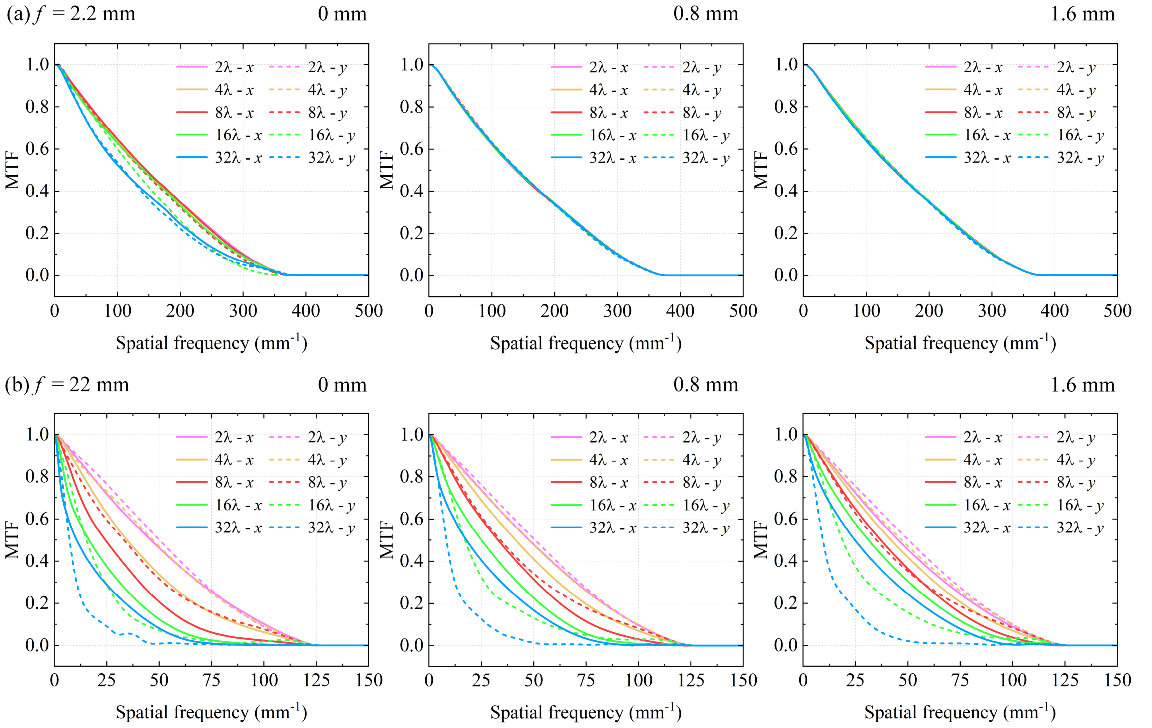

Figure S9. Modulation transfer function (MTF) curves for interlens separations of 2, 4, 8, 16, and 32 times the wavelength (λ). The MTF is evaluated at focal lengths of (a) $f = 2$ mm and (b) $f = 22$ mm, with image heights of 0, 0.8, and 1.6 mm considered at each focal length.

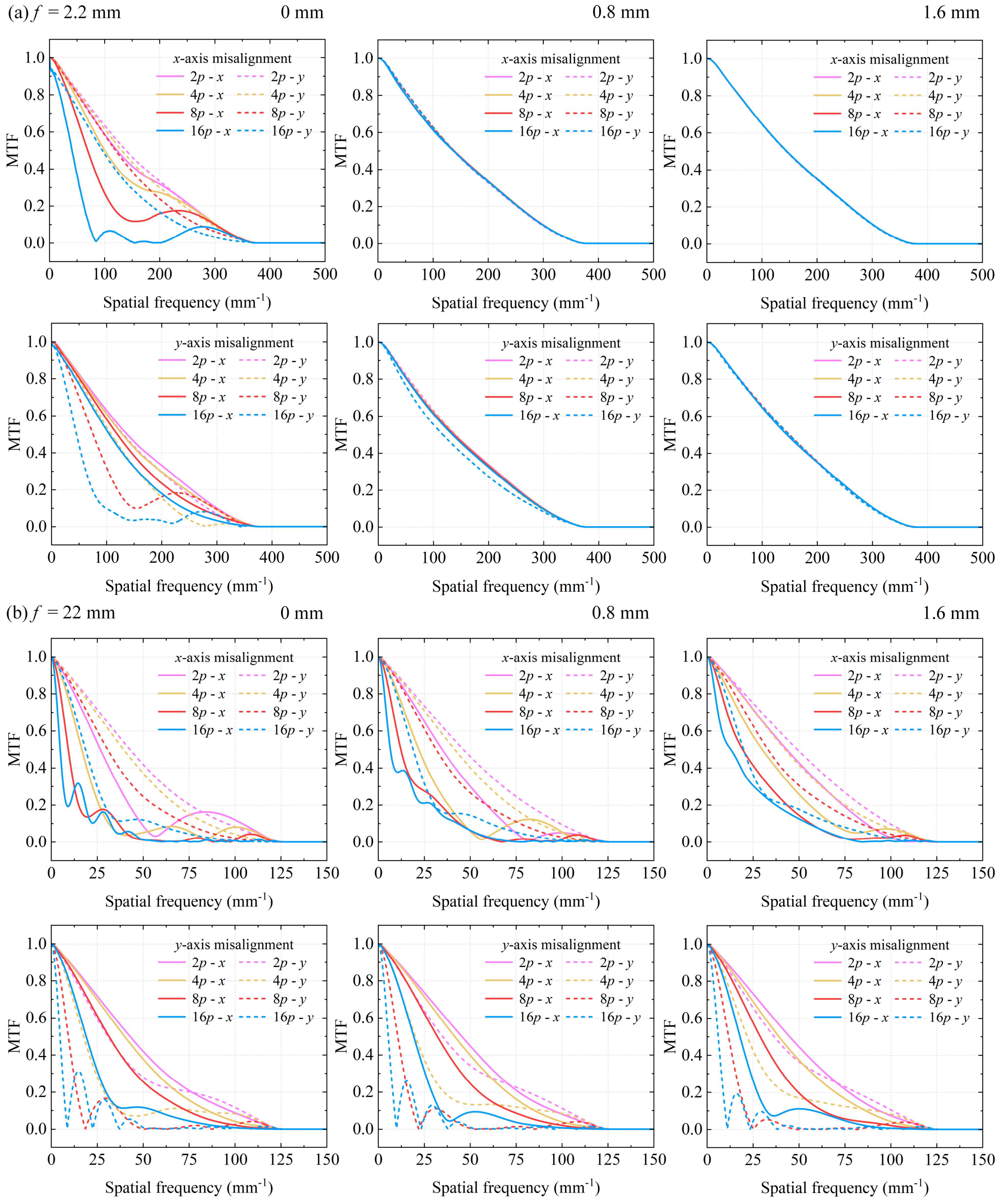

Figure S10. Modulation transfer function (MTF) curves for misalignments of 2, 4, 8, and 16 periods (p) along either the x or y direction. The MTF is evaluated at focal lengths of (a) f = 2 mm and (b) f = 22 mm , with image heights of 0, 0.8, and 1.6 mm considered at each focal length.

Reference

[1] F. Shen and A. Wang, “Fast-Fourier-transform based numerical integration method for the Rayleigh-Sommerfeld diffraction formula,” *Appl. Opt.*, vol. 45, no. 6, pp. 1102–1110, Feb. 2006, https://doi.org/10.1364/AO.45.001102.

[2] Y. Wei *et al.*, “Compact Optical Polarization-Insensitive Zoom Metalens Doublet,” *Adv. Opt. Mater.*, vol. 8, no. 13, p. 2000142, 2020, https://doi.org/10.1002/adom.202000142.

[3] Y. Qian, B. Hu, Z. Du, and J. Liu, “Reinforced design method for moiré metalens with large spacing,” *Opt. Express, OE*, vol. 29, no. 17, pp. 26496–26508, Aug. 2021, https://doi.org/10.1364/OE.431112.
